# Bayesian Multi-Plate High-Throughput Screening of Compounds

**DOI:** 10.1038/s41598-018-27531-w

**Published:** 2018-06-22

**Authors:** Ivo D. Shterev, David B. Dunson, Cliburn Chan, Gregory D. Sempowski

**Affiliations:** 1 Duke Human Vaccine Institute, Duke University, Durham, NC, 27710 USA; 2 Department of Statistical Science, Duke University, Durham, NC, 27708 USA; 3 Department of Biostatistics and Bioinformatics, Duke University, Durham, NC, 27705 USA

## Abstract

High-throughput screening of compounds (chemicals) is an essential part of drug discovery, involving thousands to millions of compounds, with the purpose of identifying candidate hits. Most statistical tools, including the industry standard B-score method, work on individual compound plates and do not exploit cross-plate correlation or statistical strength among plates. We present a new statistical framework for high-throughput screening of compounds based on Bayesian nonparametric modeling. The proposed approach is able to identify candidate hits from multiple plates simultaneously, sharing statistical strength among plates and providing more robust estimates of compound activity. It can flexibly accommodate arbitrary distributions of compound activities and is applicable to any plate geometry. The algorithm provides a principled statistical approach for hit identification and false discovery rate control. Experiments demonstrate significant improvements in hit identification sensitivity and specificity over the B-score and R-score methods, which are highly sensitive to threshold choice. These improvements are maintained at low hit rates. The framework is implemented as an efficient R extension package BHTSpack and is suitable for large scale data sets.

## Introduction

**Figure 1:**
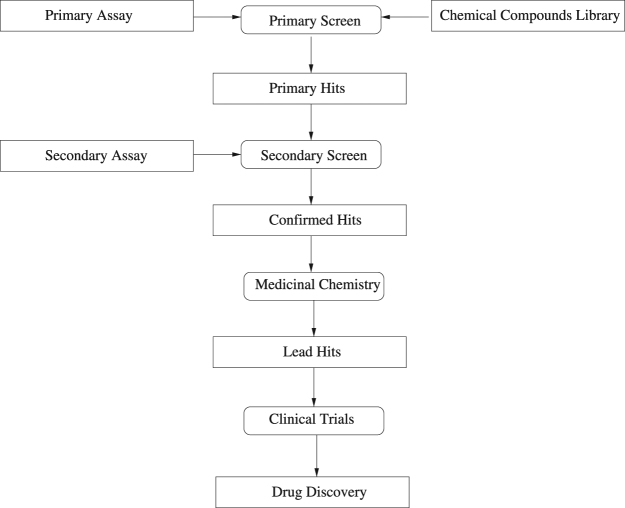
Block diagram of an HTS process.

High-throughput screening (HTS) of compounds is a critical step in drug discovery^1^. This typically involves the screening of thousands to millions of candidate compounds (chemicals). The objective is to accurately identify which compounds are candidate active compounds (hits). Those compounds will then undergo a secondary screen. A flow chart of a typical HTS process is shown in Fig. 1. The first step in the process, called primary screening, is a comprehensive scan of tens of thousands of compounds with the objective of identifying primary hits. Computational and statistical tools involved in the primary screening step need to be accurate and efficient, due to the large number of compounds to be screened.

Two types of error can occur in the primary screening process, namely false positive (FP) and false negative (FN) errors. While technological improvements and advances in experimental design and accuracy can help mitigate these two types of error, they by themselves are not able to sufficiently improve the quality of the HTS process in general and the primary screening step in particular^1^. There is a need for comprehensive statistical and computational data analysis systems that can characterize HTS data accurately and efficiently, including available prior information and borrowing information.

### HTS Data Structure

Compounds are evaluated on 96-well or 384-well plates. For 96-well plates, the first and last columns typically contain only control wells, and thus a 96-well plate only contains 80 test compounds. Similarly, the first and last columns of a 384-well plate are typically used for controls, leaving 352 wells for test compounds. We assume that each well measures a different compound activity (e.g. no replicates) and has the same concentration of compound. It is also assumed that compounds are distributed randomly within the plate.

**Figure 2:**
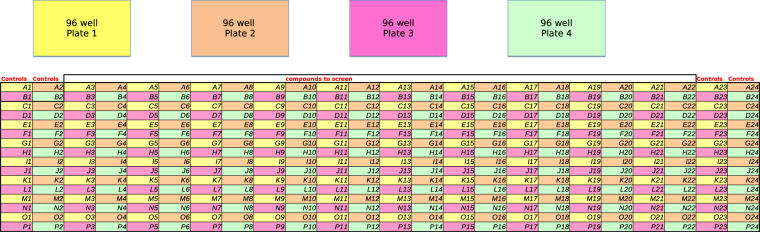
384-well plate consisting of four 96-well plates. Figure taken from^24^.

In a different 384-well plate design depicted in Fig. 2, four 96-well plates are screened as a common 384-well plate. This is done to achieve higher efficiency and number of screened compounds. Because the individual 96-well plates are processed as a whole, artifacts from robotic equipment, unintended difference in concentration, agent evaporation, or other errors^2^ might propagate through the plates. This type of cross-plate correlation is not accounted for by simple HTS systems working on individual 96-well plates.

**Figure 3:**
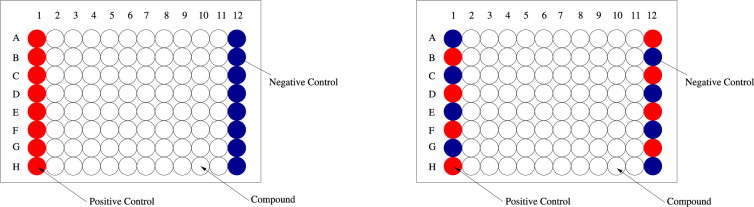
Example of a 96-well plate with compounds in the middle 80 wells and controls in the first and last column wells. Left panel shows a plate containing compounds, negative and positive controls. Right panel shows a 96-well plate in which positive and negative controls alternate to reduce plate edge effects.

Figure 3 provides a more detailed look of a 96-well plate. Ideally, controls should be placed randomly throughout the plate, to mitigate edge effects. However, the standard practice is to place the controls in the first and last columns and the compounds in inner columns.

### High-Throughput Screening of Compounds Methods

High-throughput screening statistical practice^1,3^ has traditionally used simple methods such as the B-score^4^, R-score^5^, Z-score and the normalized percent inhibition (NPI), for measuring compound activity and identifying potential candidate hits. These methods transform the compound raw value into the so called normalized value, which can then be used directly to assess compound activity. Each of the above-mentioned methods has advantages and disadvantages and they differ in terms of how controls are used. The B-score, R-score and Z-score do not use controls in the normalization process, while the NPI makes use of both positive and negative controls.

The Z-score and the NPI work on per individual compound basis. The NPI, which has a biologically plausible interpretation as the percent activity relative to an established positive control, is defined as

1 $$NPI=\frac{{z}_{p}-z}{{z}_{p}-{z}_{n}}\mathrm{100 \% },$$

where *z* is the compound raw value and *z*_*n*_ and *z*_*p*_ are the negative and positive control raw values respectively.

The Z-score is defined as

2 $$Z=\frac{z-{\mu }_{z}}{{\sigma }_{z}},$$

where *μ*_*z*_ and *σ*_*z*_ are the mean and standard deviation respectively of all compounds in the plate.

The B-score and the R-score work on a per plate basis in the sense that the plate geometry has an effect on the computed score. The B-score is defined as

3 $$B=\frac{{r}_{z}}{MA{D}_{z}},$$

where *r*_*z*_ is a matrix of residuals obtained after a median Polish fitting procedure and *MAD*_*z*_ is the median absolute deviation.

The R-score^5^ uses the robust linear model (rlm) as an alternative to the median polish, to obtain robust estimates of row and column effects. Instead of the median absolute deviation, the scale estimate from rlm is used to compute the score. The R-score has been demonstrated^5^ to outperform the B-score in a number of cases, especially when there is an absence of positional effects in the HTS data plates.

**Figure 4:**
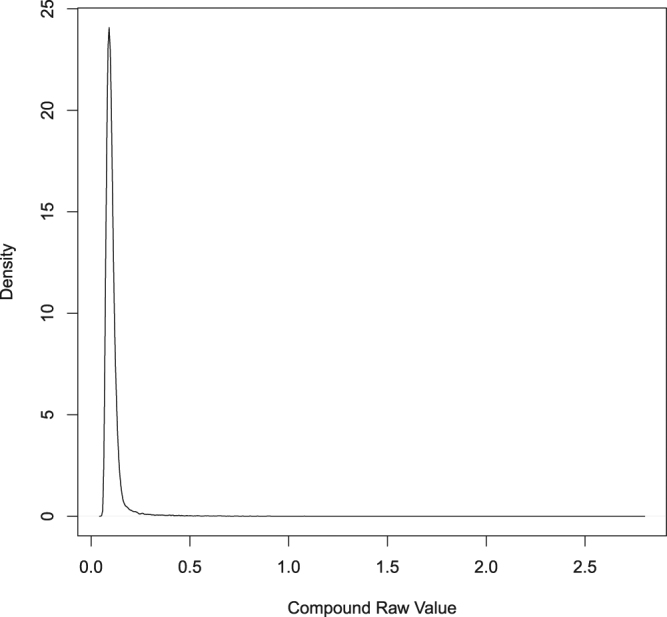
Density of mast cell activated compounds, exhibiting a log-normal law with a large positive outlier. Data are under the auspices of NIH contract No. HHSN272201400054C.

The NPI, Z-score, B-score and R-score all have limitations. The NPI is very sensitive to edge effects, since it uses the negative and positive control wells that are typically in the outer columns. The Z-score is susceptible to outliers (although this can be mitigated by an alternative called the BZ-score^5^) and assumes normally distributed compound readout values, an implausible assumption in many screening contexts (Fig. 4). Although the B-score takes into account systematic row and column plate effects and is the method of choice^1^ in many cases, it requires an arbitrary threshold (the same applies to the R-score) to identify hits and tends to miss important compounds with minimal or moderate activity. Critically, most methods treat each plate independently. In some cases, systematic experimental and plate design effects may induce correlation among groups of plates. A variation of the B-score method described in^4^ makes use of compound wells from different plates by constructing a smooth function which is then applied locally to individual wells. The assumption in their approach is that systematic plate effects are fairly spatially and temporally (across plates) consistent. It would be desirable to have a more flexible system that works on multiple plates simultaneously, while selectively sharing statistical strength of well regions across plates.

A Bayesian approach for hit selection in RNAi screens was proposed in^6^. The model imposes separate Gaussian priors on active, inactive and inhibition siRNAs. Inference is based on hypothesis testing via posterior distributions. The posterior distributions are then directly used to control false discovery rate (FDR)^7^. The proposed method is parametric and although it may be reasonable in some cases to model the siRNAs as normally distributed, many data in practice and particularly HTS data are not Gaussian (as shown in Fig. 4). Additionally, the priors of the proposed method incorporate common information that is pooled from all plates. This type of information sharing is fixed and is different from the multi-plate sharing mechanism in machine learning, where different groups of data iteratively and selectively share information via a global layer^8^.

In this paper we develop a new system for HTS of compounds based on Bayesian nonparametric modeling. The nonparametric method does not use controls and is capable of characterizing HTS data that are not necessarily Gaussian distributed. It can handle multiple plates simultaneously and is able to selectively share statistical strength among plates. This selective sharing mechanism is important for discovering systematic experimental effects that propagate differently among plates. We develop an efficient Markov chain Monte Carlo (MCMC) sampler for estimating the compound readout posteriors. Based on posterior probabilities specifying if a compound is active or not, it is possible to determine probabilistic significance, control FDR^9^ and adjust for multiple comparison^10^ in a Bayesian hierarchical manner. The framework is implemented as an R extension package BHTSpack^11^.

## Materials and Methods

### Statistical Model

Dirichlet process Gaussian mixtures (DPGM)^12,13^ constitute a powerful class of nonparametric models that can describe a wide range of distributions encountered in practice. The simplicity of DPGM and their ease of implementation have made them a preferable choice in many applications, as well as building blocks of more complex models and systems. In the DPGM framework, the Dirichlet process (DP)^14,15^ models the mixing proportions of the Gaussian components. The hierarchical Dirichlet process (HDP)^8^ is particularly suitable for modeling multi-task problems in machine learning. An example of multi-task learning is the simultaneous segmentation of multiple images (tasks) for the purpose of image analysis, which can be facilitated by the use of the HDP or a variation of it^16,17^. The analogy is that while in image analysis the input data are images of pixel intensities, in our HTS of compounds scenario they are plates of compound readouts. However, spatially proximate pixels in a typical image are correlated, while compounds in a plate are ideally (in the absence of effects) unrelated.

Our framework deploys two HDPs to characterize the active and inactive components. In the following sequel we approximate the DP via the finite stick-breaking representation^18^. Let *m* ∈ {1, …, *M*} denote the plate index, where *M* is the total number of plates. Let *i* ∈ {1, …, *n*_*m*_} denote the compound well index within a plate, *h* ∈ {1, …, *H*} denote the DP mixture cluster index within a plate and *k* ∈ {1, …, *K*} denote the global DP component index. Let superscripts (1) and (0) refer to active and inactive compounds, respectively. Motivated in part by^19^, we propose to model the compound activity via a two HDP mixture model as follows:

$${z}_{mi}\sim \pi \sum _{h=1}^{H}{\lambda }_{mh}^{\mathrm{(1)}}{\mathscr{K}}({z}_{mi};{\theta }_{h}^{\mathrm{(1)}})+(1-\pi )\sum _{h=1}^{H}{\lambda }_{mh}^{\mathrm{(0)}}{\mathscr{K}}({z}_{mi};{\theta }_{h}^{\mathrm{(0)}})$$

where *z*_*mi*_ is the measured activity of the *i*th compound from plate *m* and ${\mathscr{K}}(\,\cdot ;\theta )$ is a Gaussian kernel with parameters *θ*.

Continuing the HDP model specification, we have:

4 $$({\lambda }_{m1}^{(1)},\ldots ,{\lambda }_{mH}^{(1)})={G}_{m}^{(1)}\sim \mathrm{DP}({\alpha }_{1},{\lambda }_{H}^{(1)})$$

5 $$({\lambda }_{m1}^{(0)},\ldots ,{\lambda }_{mH}^{(0)})={G}_{m}^{(0)}\sim \mathrm{DP}({\alpha }_{0},{\lambda }_{H}^{(0)})$$

6 $$({\lambda }_{1}^{(1)},\ldots ,{\lambda }_{K}^{(1)})={G}_{0}^{(1)}\sim \mathrm{DP}({\tau }_{1},{\lambda }_{K}^{(1)})$$

7 $$({\lambda }_{1}^{(0)},\ldots ,{\lambda }_{K}^{(0)})={G}_{0}^{(0)}\sim \mathrm{DP}({\tau }_{0},{\lambda }_{K}^{(0)})$$

where *DP*(*α*, ⋅) denotes the finite stick-breaking representation of the DP with concentration parameter *α* (see supplementary materials for more details about the stick-breaking construction).

The beta distribution, with its sub-case the uniform distribution, is defined on the interval [0, 1] and is flexible enough to model mixing proportions. At the same time, the gamma distribution, a conjugate (its posterior is also gamma) to the beta distribution, has a support (0, +∞) and is particularly suitable for specifying DP concentration parameters, adding flexibility to the overall model. We therefore place a beta prior on the HDP mixing proportion, and gamma priors on the DP concentrations:

8 $$\pi \sim {\rm{Beta}}({a}_{\pi },{b}_{\pi })$$

9 $${\alpha }_{1},{\alpha }_{0}\sim {\rm{Ga}}({a}_{\alpha },{b}_{\alpha })$$

10 $${\tau }_{1},{\tau }_{0}\sim {\rm{Ga}}({a}_{\tau },{b}_{\tau })$$

Finally, we place Normal-inverse gamma priors on the Gaussian kernel parameters:

11 $${\theta }_{h}^{(1)}\sim {\mathscr{N}}({\mu }_{1}|{\mu }_{10},{\sigma }_{1}^{2}){\rm{I}}{\rm{n}}{\rm{v}}-{\rm{G}}{\rm{a}}({\sigma }_{1}^{2}|a,b)$$

12 $${\theta }_{h}^{(0)}\sim {\mathscr{N}}({\mu }_{0}|{\mu }_{00},{\sigma }_{0}^{2}){\rm{I}}{\rm{n}}{\rm{v}}-{\rm{G}}{\rm{a}}({\sigma }_{0}^{2}|a,b)$$

where $\{{\mu }_{1},{\sigma }_{1}^{2}\}$ and $\{{\mu }_{0},{\sigma }_{0}^{2}\}$ model the activity mean and variance of active and inactive compounds respectively. The hyperparameters *μ*_11_ and *μ*_10_ reflect our prior belief for the expected activity level of active and inactive compounds, respectively. These hyperparameters do not need to be precisely specified and the next subsection provides guidelines for choosing appropriate values.

At each iteration of the MCMC sampler, the model determines plate-specific groups of active and inactive compounds, via the local DP layers ${G}_{m}^{(1)}$ and ${G}_{m}^{(0)}$ respectively. A group of compounds (either active or inactive) will share the same DP mixture component variables. At the same time, the model also clusters the plate-specific groups of compounds into global groups of active and inactive compounds, via the global DP layers ${G}_{0}^{(1)}$ and ${G}_{0}^{(0)}$ respectively. A global group of compounds may contain compounds from different plates that share the same global DP mixture variables. This selective sharing mechanism of the HDP framework mitigates systematic experimental effects that propagate differently among plates and plate regions.

### Specification of hyperparameters {*μ*_10_, *μ*_00_, *a*, *b*}

The compound data mean and variance can be used to specify the model hyperparameters {*μ*_10_, *μ*_00_, *a*, *b*}, without the help from controls. Let *μ* denote the mean of the compound data. We found that automatically setting *μ*_00_ = 0.5 *μ* and *μ*_10_ = 3 *μ*_00_ yields good results across cases in our experiments.

The compound variance hyperparameters {*a*, *b*} are common for both active and inactive compounds, but the model facilitates different active ${\sigma }_{1}^{2}$ and inactive ${\sigma }_{0}^{2}$ compound variances. These hyperparameters can be derived using the compound data, and the expressions for the inverse gamma mean $\frac{b}{a-1}$ (for $a > 1$) and variance $\frac{{b}^{2}}{{(a-1)}^{2}(a-2)}$ (for $a > 2$). Let *v* denote the compound data variance. The choice of the specific value for inverse gamma variance determines the concentration of the prior around the variance of the compound data. The smaller the value the more concentrated the prior is. We found that the value 10^−4^ gave a well concentrated prior with a valid hyperparameter $a > 2$. As a result, setting the inverse gamma variance to 10^−4^ and using *v* as the mean, the hyperparameters can be explicitly derived as:

13 $$a=\frac{{v}^{2}}{{10}^{-4}}+2,$$

14 $$b=\frac{{v}^{3}}{{10}^{-4}}+v.$$

**Figure 5:**
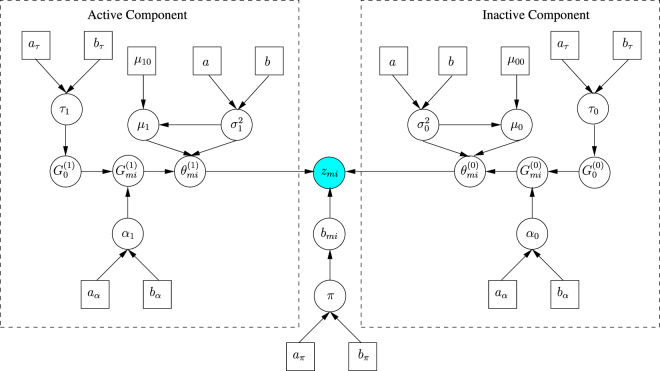
A graphical representation of BHTS model. The blue circle represents the observed variable, white circles represent hidden (latent) variables and squares represent hyper-parameters. Conditional dependence between variables is shown via the directed edges. The latent binary variable *b*_*mi*_ specifies active (1) or inactive (0) compound.

A graphical representation of the complete BHTS model is shown in Fig. 5.

We derive MCMC update equations based on approximate full conditional posteriors of the model parameters and construct a Gibbs sampler that iteratively samples from these update equations. The stick breaking construction^18^ is used in the approximate posterior distributions of the global and local DP weights. The update equations are shown in the supplementary materials.

### False Discovery Rate and Multiplicity Correction

Our problem can be formulated as performing ${\sum }_{m}{n}_{m}$ dependent hypothesis tests of *b*_*mi*_ = 0 versus *b*_*mi*_ = 1. Following^20^, an estimate to FDR for a given threshold *r* can be computed as:

15 $$\overline{\,{\rm{FDR}}}(r)=\frac{\sum _{m,i}\mathrm{1(}\hat{\pi }({z}_{mi}) > r\mathrm{)(1}-\hat{\pi }({z}_{mi}))}{\sum _{m,i}\mathrm{1(}\hat{\pi }({z}_{mi}) > r)},$$

where $\hat{\pi }({z}_{mi})$ is the posterior probability estimate of the compound *z*_*mi*_ being a hit (see supplementary) and 1(⋅) is the indicator function. Since the model is fully Bayesian, multiple comparison is automatically accounted for^10^ in the estimated posteriors $\hat{\pi }({z}_{mi})$.

## Results

We assess the performance of the BHTS method using synthetically generated and real data sets, and compare it with the B-score and R-score methods in terms of receiver operating characteristic (ROC) curves and area under the curve (AUC). We also perform experiments with a data set containing very low proportion of compound hits. The R extension packages sights^21^ (implementing the R-score) and pROC^22^ were employed in the analysis.

### Synthetic Compound Data

We constructed synthetic data for the purpose of assessing sensitivity and specificity of the proposed algorithm. Here we describe synthetic data generation based on the 96-well plate format. Experiments with 384-well plates are described and presented in the supplementary. We generated a set of 80 × 10^3^ compounds consisting of hits and non-hits. The hits were generated from a four component log-normal mixture model with means {0.20, 0.24, 0.28, 0.32} and variances {0.0020, 0.0022, 0.0024, 0.0026}. Similarly, the non-hits were generated from a four component log-normal distribution with means {0.10, 0.12, 0.14, 0.16} and variances {0.010, 0.011, 0.012, 0.013}. The compounds were then randomly distributed among 1000 compound plates, with each plate consisting of eight rows and ten columns. In choosing the means and variances we tried to obtain a synthetic data that will closely resemble the real compound data shown in Fig. 4.

**Figure 6:**
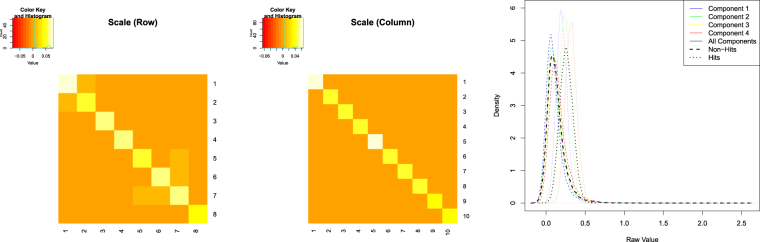
Synthetic compound data used in the experiments. Left and middle plots show scale matrices used in generating the synthetic noise plates, indicating predominantly row dependent within plate effects. Right plot shows density of resulting synthetic compound and plate effect data.

We simulated plate effects by generating random noise from the matrix-normal distribution, with a zero location matrix and specific row and column scale matrices. The row and column scale matrices were designed in a way to reflect the structure of within plate row and column effects encountered in practice. Specifically, we used a real data set of compounds exhibiting the plate design shown in Fig. 3. We excluded the control well columns and computed B-scores based on individual 8 × 10 compound well plates. We then estimated the row-wise and column-wise covariance matrices of the difference between the compound raw values and their B-scores. The estimated covariance matrices were then properly scaled and used as row and column scale matrices (shown in Fig. 6) in generating the plate noise effects. An independently drawn noise plate was added to each of the compound plates. The resulting data plates were used as test data.

Experiments were performed with data sets containing different proportions of active and inactive compounds. Considering the fact that a large collection of compounds will probably contain a relatively small number of candidate compound hits of interest, we experimented with data sets containing 10%, 5%, and 1% of active compounds, respectively. See supplementary materials for specific choice of model hyperparameter values.

### Comparison with B-score and R-score Methods

**Figure 7:**
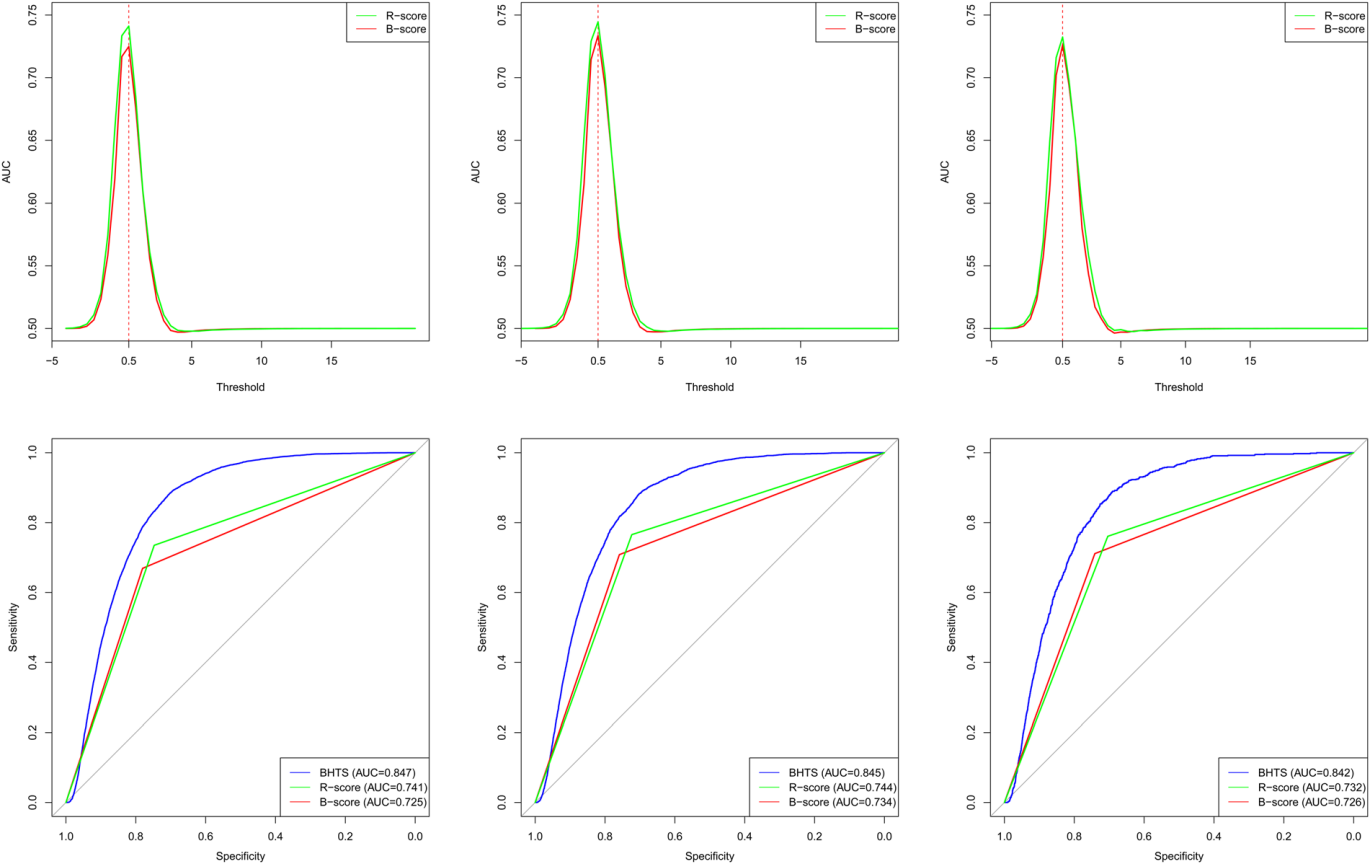
Top row shows AUC plots of B and R score methods, as functions of thresholds. Bottom row shows ROC plots of the B-score, R-score and BHTS methods. Data sets containing 10% (left column), 5% (middle column) and 1% (right column) of active compounds, respectively. The piecewise-linear shape of the B and R score curves is due to the binary nature of the predictor.

Experimental ROC results are shown in Fig. 7. The B and R score ROC curves which have piecewise-linear shape due to the binary nature of the predictor, are based on the maximum achievable AUC threshold. It can be seen that the BHTS method improves upon the other methods in terms of classification accuracy. The results in Fig. 7 also demonstrate that the B-score is highly sensitive to a particularly chosen optimal threshold, as evidenced by the spike in the AUC curve as a function of the threshold.

### Hyperparameter Sensitivity Analysis

**Figure 8:**
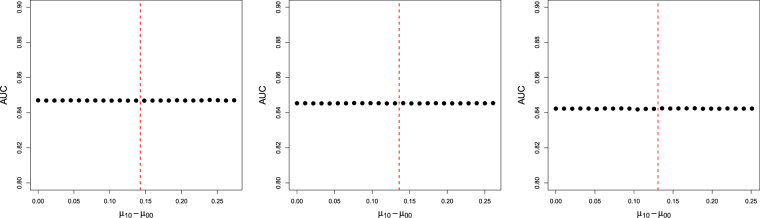
AUC as a function of (*μ*_10_ − *μ*_00_), for data sets containing 10% (left plot), 5% (middle plot) and 1% (right plot) of active compounds. Red line indicates mean of compound data.

In this subsection we assess the sensitivity of the proposed method to the choice of hyperparameter values {*μ*_10_, *μ*_00_} by computing the AUC for a range of values of the difference (*μ*_10_ − *μ*_00_). Experimental results are shown in Fig. 8. It can be seen that the model performs similarly in terms of AUC, for a range of (*μ*_10_ − *μ*_00_) values and different data sets.

### Low Hit Rates

We assess the proposed method capability to identify potential hits from data sets containing very small proportion of hits. Low hit rates of HTS have been reported in^23^. In their paper, the authors describe a corporate library of approximately 4 × 10^5^ compounds that was screened for compound hits that inhibited PTP1B. Of approximately 4 × 10^5^ molecules tested, 85 (a hit rate of 0.021%) inhibited the enzyme with IC_50_ values less than 100 *μ*M.

**Figure 9:**
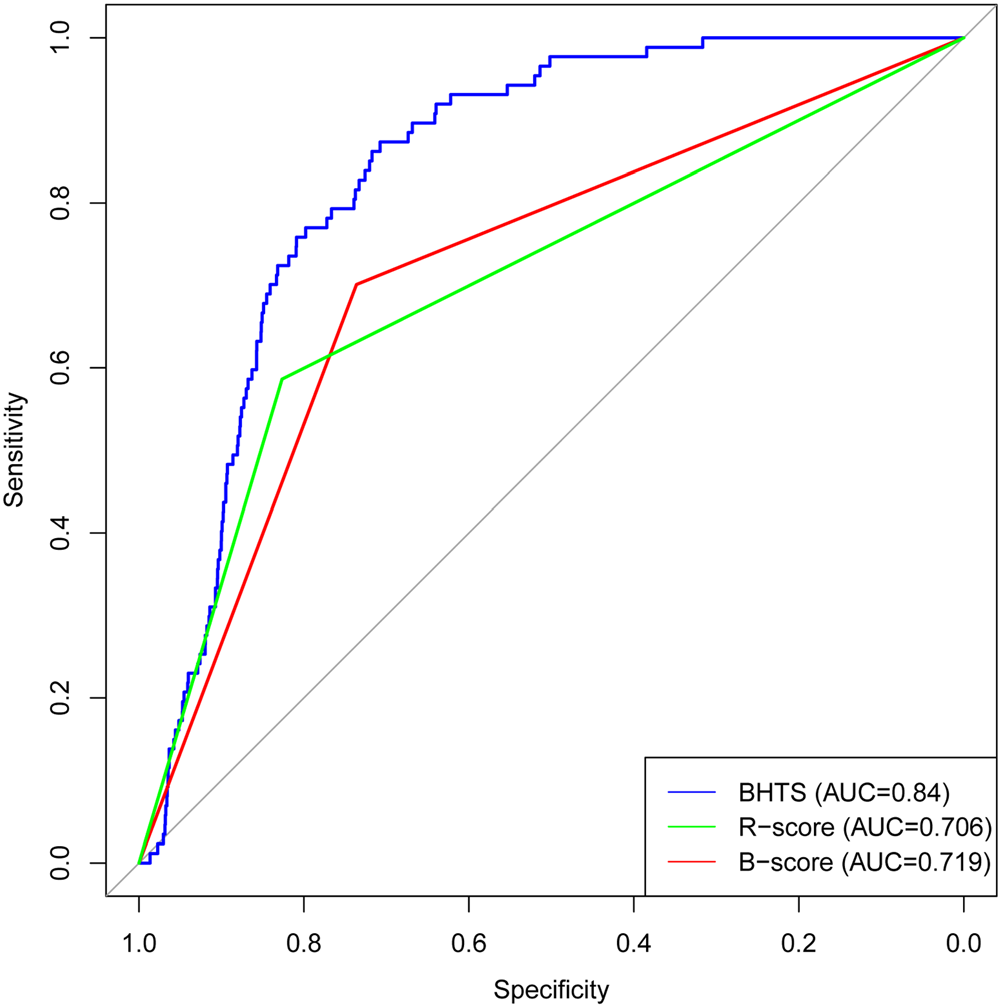
ROC plots of the B-score, R-score and BHTS methods. Data set containing 0.021% of active compounds.

The above mentioned corporate library of compounds is not publicly available. To simulate such a low hit rate scenario, we generated 4 × 10^5^ compounds in the same way as before, but this time the compound data set contained 0.021% of active compounds. Experimental ROC results are shown in Fig. 9. It can be seen that the BHTS method maintains its performance improvement in the case of low hit rates.

### Real Compound Data

We considered the data set “Inhibit E.coli Cell Division via Induction of Filamentation, but Excluding Filamentation Induced by DNA Damage”, available from ChemBank (http://chembank.broadinstitute.org) at Broad Institute. The data set consisted of twenty four assays, most of which were run on 352-well (excluding controls) plates. From all screened compounds, 389 were confirmed hits. The data set was collected under different experimental conditions, namely three organisms (DRC39, DRC40, DRC41) and two incubation times (24 h and 48 h). We therefore restricted the analysis to one organism and incubation time (DRC39 at 24 h). Considering only 352-well format plates, we had 57 plates available for the analysis. The plates contained data from 4 assays. The plates were run in duplicates, but only one of the replicates was present for each compound well. We therefore used that replicate in the subsequent analysis. Since compounds were screened in more than one assay, the number of compound hit wells was 434 out of a total number of 352 × 57 = 20, 064 wells.

**Figure 10:**
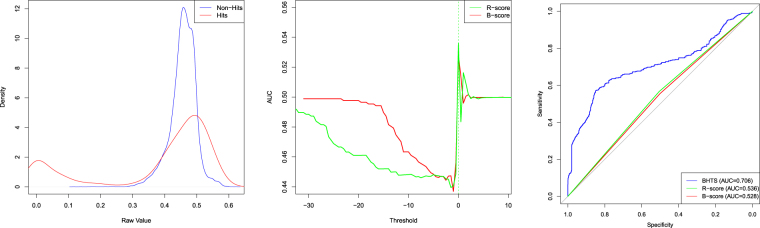
Real compound data and experimental results. Raw compound data density (left), AUC plots (middle) and ROC curves (right).

Prior to applying the BHTS, B-score and R-score methods, we normalized the data so that each plate had a mean zero and variance one (can be seen as computing the Z-score). Experimental results, along with the compound density (being quite idiosyncratic), are shown in Fig. 10. It can be seen from the ROC curves that this real compound data set is far more challenging than the considered synthetic data set examples, but the BHTS method still outperforms the B-score and R-score methods.

### Implementation and Scalability

We implemented the proposed model as an R package BHTSpack^11^, with some of the inner routines implemented in C/C++. We experimented on a laptop with an Intel(R) Core(TM) i7-4600M CPU @ 2.90 GHz and 8 GB of RAM, running the 64-bit Ubuntu Linux operating system. It takes 30 minutes to complete 7 × 10^3^ Gibbs sampler iterations, using 10^3^ plates with 80 × 10^3^ compounds. Additionally, it takes 23 hours to complete the same number of iterations, using 50 × 10^3^ plates with 4 × 10^6^ compounds. The proposed MCMC algorithm for Bayesian posterior computation had good mixing rates across cases in our experiments (see trace and autocorrelation function plots in the supplementary materials).

## Discussion

We developed a new probabilistic framework for primary hit screening of compounds based on Bayesian nonparametric modeling. The statistical model is capable of simultaneously identifying hits from multiple plates, with possibly different numbers of unique compounds, and without the use of controls. It selectively shares statistical strength among different regions of plates, thus being able to characterize systematic experimental effects across plate groups. The nonparametric nature of the model makes it suitable for handling real compound data that are not necessarily Gaussian distributed. The probabilistic hit identification rules of the algorithm facilitate principled statistical hit identification and FDR control. Experimental validation with synthetic and real compound data show improved sensitivity and specificity over the B-score and R-score methods, which are shown to be highly sensitive to the choice of an optimal threshold. The performance improvement of the BHTS method is shown to be maintained at low hit rates. An efficient implementation in the form of an R extension package BHTSpack^11^ makes the method applicable to large scale HTS data analysis.

## Electronic supplementary material

Supplementary Information
