# Supplementary material for: Bayesian Multi-Plate High-Throughput Screening of Compounds
Source: Sci Rep. 2018 Jun 22;8:9551. doi: 10.1038/s41598-018-27531-w (PMC6015058; doi:10.1038/s41598-018-27531-w)
Supplement: Supplementary file 1 — Supplementary Information [file 41598_2018_27531_MOESM1_ESM.pdf]

Bayesian Multi-Plate High-Throughput Screening of Compounds: Supplementary Material

Ivo D. Shterev*[1], David B. Dunson[2], Cliburn Chan[3], and Gregory D. Sempowski[1]

[1]Duke Human Vaccine Institute, Duke University
[2]Department of Statistical Science, Duke University
[3]Department of Biostatistics and Bioinformatics, Duke University

April 12, 2018

A Finite Stick-Breaking DP Representation

This section provides a specification of the finite stick-breaking representation [1] of the global $\{\boldsymbol{\lambda_K^{(1)}}, \boldsymbol{\lambda_K^{(0)}}\}$ and local $\{\boldsymbol{\lambda_H^{(1)}}, \boldsymbol{\lambda_H^{(0)}}\}$ DP weight priors.

$$
\begin{aligned}
\boldsymbol{\lambda_K^{(1)}} &= (\lambda_1^{(1)}, \ldots, \lambda_K^{(1)}), && (1)\\
\lambda_k^{(1)} &= \nu_k^{(1)} \prod_{l=1}^{k-1} (1 - \nu_l^{(1)}), \text{ for } k \in \{1, \ldots, K\} && (2)\\
\nu_k^{(1)} &\sim \text{Beta}(1, \tau_1), \text{ for } k \in \{1, \ldots, K\} && (3)\\
\boldsymbol{\lambda_K^{(0)}} &= (\lambda_1^{(0)}, \ldots, \lambda_K^{(0)}), && (4)\\
\lambda_k^{(0)} &= \nu_k^{(0)} \prod_{l=1}^{k-1} (1 - \nu_l^{(0)}), \text{ for } k \in \{1, \ldots, K\} && (5)\\
\nu_k^{(0)} &\sim \text{Beta}(1, \tau_0), \text{ for } k \in \{1, \ldots, K\} && (6)\\
\boldsymbol{\lambda_H^{(1)}} &= (\lambda_1^{(1)}, \ldots, \lambda_H^{(1)}), && (7)\\
\lambda_h^{(1)} &= \nu_h^{(1)} \prod_{l=1}^{h-1} (1 - \nu_l^{(1)}), \text{ for } h \in \{1, \ldots, H\} && (8)\\
\nu_h^{(1)} &\sim \text{Beta}(1, \alpha_1), \text{ for } h \in \{1, \ldots, H\} && (9)\\
\boldsymbol{\lambda_H^{(0)}} &= (\lambda_1^{(0)}, \ldots, \lambda_H^{(0)}), && (10)\\
\lambda_h^{(0)} &= \nu_h^{(0)} \prod_{l=1}^{h-1} (1 - \nu_l^{(0)}), \text{ for } h \in \{1, \ldots, H\} && (11)\\
\nu_h^{(0)} &\sim \text{Beta}(1, \alpha_0), \text{ for } h \in \{1, \ldots, H\}. && (12)
\end{aligned}
$$

*Corresponding Author: Ivo D. Shterev, PhD; email: i.shterev@duke.edu

B MCMC Update Equations

This section presents the MCMC update equations based on approximate full conditional posteriors of the model parameters and construct a Gibbs sampler that iteratively samples from these update equations.

Let indicator variable $I_{mi}^{(1)} \in \{1, \ldots, K\}$ specify the active component to which the ith compound from the mth plate belongs. Analogously, $I_{mi}^{(0)} \in \{1, \ldots, K\}$ specifies the inactive component to which the ith compound from the mth plate belongs.

- updating compound hit indicator variable b_{mi}.

$$p(b_{mi}|\hat{\pi}(z_{mi})) \propto \text{Bernoulli}(b_{mi}|\hat{\pi}(z_{mi})), \tag{13}$$

where

$$\hat{\pi}(z_{mi}) = \frac{1}{1 + \frac{1-\pi}{\pi} \frac{\sum_h \lambda_{mh}^{(0)} \mathcal{K}(z_{mi}; \theta_h^{(0)})}{\sum_h \lambda_{mh}^{(1)} \mathcal{K}(z_{mi}; \theta_h^{(1)})}}. \tag{14}$$

- updating π.

$$p(\pi|\text{all } b_{mi}) = \text{Beta}(\pi|\hat{a}_\pi, \hat{b}_\pi), \tag{15}$$

where

$$\hat{a}_\pi = a_\pi + \sum_{m,i} b_{mi}, \tag{16}$$

$$\hat{b}_\pi = b_\pi + \sum_{m,i} (1 - b_{mi}). \tag{17}$$

- updating $\theta_k^{(1)} = \{\mu_{1k}, \sigma_{1k}^2\}$

$$p(\mu_{1k}, \sigma_{1k}^2|z_{mi}) = \mathcal{N}\Big(\mu_{1k}\Big|\frac{\mu_{10} + n_k^{(1)} \bar{z}_k^{(1)}}{n_k^{(1)} + 1}, \frac{\sigma_{1k}^2}{n_k^{(1)} + 1}\Big)\text{Inv-Ga}\Big(\sigma_{1k}^2\Big|\hat{a}_{1k}, \hat{b}_{1k}\Big), \tag{18}$$

$$\tag{19}$$

where

$$\hat{a}_{1k} = a + n_k^{(1)} \tag{20}$$

$$\hat{b}_{1k} = b + 0.5 \sum_{m,i} (z_{mi} - \bar{z}_k^{(1)})^2 + 0.5 \frac{n_k^{(1)}}{n_k^{(1)} + 1} (\mu_{10} - \bar{z}_k^{(1)})^2 \tag{21}$$

$$\bar{z}_k^{(1)} = \frac{1}{n_k^{(1)}} \sum_{m,i} b_{mi} 1(I_{mi}^{(1)} = k) z_{mi} \tag{22}$$

$$n_k^{(1)} = \sum_{m,i} b_{mi} 1(I_{mi}^{(1)} = k) \tag{23}$$

$$1(I_{mi}^{(1)} = k) = \begin{cases} 1 & \text{if } z_{mi} \text{ belongs to the } k\text{th active component,} \\ 0 & \text{if otherwise.} \end{cases} \tag{24}$$

- updating $\theta_k^{(0)} = \{\mu_{0k}, \sigma_{0k}^2\}$

$$p(\mu_{0k}, \sigma_{0k}^2 | z_{mi}) = \mathcal{N}\Big(\mu_{0k}\Big|\frac{\mu_{10} + n_k^{(0)}\bar{z}_k^{(0)}}{n_k^{(0)}+1}, \frac{\sigma_{0k}^2}{n_k^{(0)}+1}\Big)\text{Inv-Ga}\Big(\sigma_{0k}^2\Big|\hat{a}_{0k}, \hat{b}_{0k}\Big), \tag{25}$$

$$\tag{26}$$

where

$$\hat{a}_{0k} = a + n_k^{(0)} \tag{27}$$

$$\hat{b}_{0k} = b + 0.5\sum_{m,i}(z_{mi} - \bar{z}_k^{(0)})^2 + 0.5\frac{n_k^{(0)}}{n_k^{(0)}+1}(\mu_{00} - \bar{z}_k^{(0)})^2 \tag{28}$$

$$\bar{z}_k^{(0)} = \frac{1}{n_k^{(0)}}\sum_{m,i}(1-b_{mi})1(I_{mi}^{(0)} = k)z_{mi} \tag{29}$$

$$n_k^{(0)} = \sum_{m,i}(1-b_{mi})1(I_{mi}^{(0)} = k) \tag{30}$$

$$1(I_{mi}^{(0)} = k) = \begin{cases} 1 & \text{if } z_{mi} \text{ belongs to the } k\text{th inactive component,} \\ 0 & \text{if otherwise.} \end{cases} \tag{31}$$

- updating $\lambda_{mh}^{(1)}$.

$$\lambda_{mh}^{(1)} = \nu_{mh}^{(1)}\prod_{l=1}^{h-1}(1-\nu_{ml}^{(1)}) \tag{32}$$

$$\nu_{mh}^{(1)} \sim \text{Beta}(1 + p_{mh}^{(1)}, \alpha_1 + \sum_{l=h+1}^{H} p_{ml}^{(1)}) \tag{33}$$

$$p_{mh}^{(1)} = \sum_i b_{mi}1(J_{mi}^{(1)} = h) \tag{34}$$

$$1(J_{mi}^{(1)} = h) = \begin{cases} 1 & \text{if } z_{mi} \text{ belongs to the } h\text{th active cluster,} \\ 0 & \text{if otherwise.} \end{cases} \tag{35}$$

- updating $\lambda_{mh}^{(0)}$.

$$\lambda_{mh}^{(0)} = \nu_{mh}^{(0)}\prod_{l=1}^{h-1}(1-\nu_{ml}^{(0)}) \tag{36}$$

$$\nu_{mh}^{(0)} \sim \text{Beta}(1 + p_{mh}^{(0)}, \alpha_0 + \sum_{l=h+1}^{H} p_{ml}^{(0)}) \tag{37}$$

$$p_{mh}^{(0)} = \sum_i (1-b_{mi})1(J_{mi}^{(0)} = h) \tag{38}$$

$$1(J_{mi}^{(0)} = h) = \begin{cases} 1 & \text{if } z_{mi} \text{ belongs to the } h\text{th inactive cluster,} \\ 0 & \text{if otherwise.} \end{cases} \tag{39}$$

- updating $\lambda_k^{(1)}$.

$$\lambda_k^{(1)} = \nu_k^{(1)}\prod_{l=1}^{k-1}(1-\nu_l^{(1)}) \tag{40}$$

$$\nu_k^{(1)} \sim \text{Beta}(1 + p_k^{(1)}, \tau_1 + \sum_{l=k+1}^{K} p_l^{(1)}) \tag{41}$$

$$p_k^{(1)} = \sum_{m,h} 1(J_{mh}^{(1)} = k) \tag{42}$$

$$1(J_{mh}^{(1)} = k) = \begin{cases} 1 & \text{if cluster } h \text{ belongs to the } k\text{th active component,} \\ 0 & \text{if otherwise.} \end{cases} \tag{43}$$

- updating $\lambda_k^{(0)}$.

$$\lambda_k^{(0)} = \nu_k^{(0)} \prod_{l=1}^{k-1} (1 - \nu_l^{(0)}) \tag{44}$$

$$\nu_k^{(0)} \sim \text{Beta}(1 + p_k^{(0)}, \tau_0 + \sum_{l=k+1}^{K} p_l^{(0)}) \tag{45}$$

$$p_k^{(0)} = \sum_{m,h} 1(J_{mh}^{(0)} = k) \tag{46}$$

$$1(J_{mh}^{(0)} = k) = \begin{cases} 1 & \text{if cluster } h \text{ belongs to the } k\text{th inactive component,} \\ 0 & \text{if otherwise.} \end{cases} \tag{47}$$

- updating α_1.

$$p(\alpha_1 | \text{all } \nu_{mh}^{(1)}) \propto \text{Ga}\Big(\alpha_1 | a_\alpha + M(H-1), b_\alpha - \sum_m \sum_{h=1}^{H-1} \ln(1 - \nu_{mh}^{(1)})\Big) \tag{48}$$

- updating α_0.

$$p(\alpha_0 | \text{all } \nu_{mh}^{(0)}) \propto \text{Ga}\Big(\alpha_0 | a_\alpha + M(H-1), b_\alpha - \sum_m \sum_{h=1}^{H-1} \ln(1 - \nu_{mh}^{(0)})\Big) \tag{49}$$

- updating τ_1.

$$p(\tau_1 | \text{all } \nu_k^{(1)}) \propto \text{Ga}\Big(\tau_1 | a_\tau + K - 1, b_\tau - \sum_{k=1}^{K-1} \ln(1 - \nu_k^{(1)})\Big) \tag{50}$$

- updating τ_0.

$$p(\tau_0 | \text{all } \nu_k^{(0)}) \propto \text{Ga}\Big(\tau_0 | a_\tau + K - 1, b_\tau - \sum_{k=1}^{K-1} \ln(1 - \nu_k^{(0)})\Big) \tag{51}$$

- updating $J_{mi}^{(1)}$.

$$J_{mi}^{(1)} \sim \text{Mult}(\lambda_{m1}^{(1)} \mathcal{K}(z_{mi}; \theta_{J_{m1}}^{(1)}), \ldots, \lambda_{mH}^{(1)} \mathcal{K}(z_{mi}; \theta_{J_{mH}}^{(1)})) \tag{52}$$

- updating $J_{mi}^{(0)}$.

$$J_{mi}^{(0)} \sim \text{Mult}(\lambda_{m1}^{(0)} \mathcal{K}(z_{mi}; \theta_{J_{m1}}^{(0)}), \ldots, \lambda_{mH}^{(0)} \mathcal{K}(z_{mi}; \theta_{J_{mH}}^{(0)})) \tag{53}$$

- updating $J_{mh}^{(1)}$.

$$J_{mh}^{(1)} \sim \text{Mult}(\lambda_1^{(1)} \prod_i \mathcal{K}(z_{mi}; \theta_1^{(1)})^{1(J_{mi}=h)}, \ldots, \lambda_K^{(1)} \prod_i \mathcal{K}(z_{mi}; \theta_K^{(1)})^{1(J_{mi}=h)}) \tag{54}$$

- updating $J_{mh}^{(0)}$.

$$J_{mh}^{(0)} \sim \text{Mult}(\lambda_1^{(0)} \prod_i \mathcal{K}(z_{mi}; \theta_1^{(0)})^{1(J_{mi}=h)}, \ldots, \lambda_K^{(0)} \prod_i \mathcal{K}(z_{mi}; \theta_K^{(0)})^{1(J_{mi}=h)}), \tag{55}$$

where Mult($\cdot$) denotes the multinomial distribution. We have relaxed the notation by allowing the indicator variable J to specify both data points (compounds) and cluster indicators, depending on the subscript[1]. We also drop the superscript of J where it is clear from the context.

C Choice of Hyperparameters

In these experiments, gamma density hyperparameters were fixed as $a_\alpha = a_\tau = 10$ and $b_\alpha = b_\tau = 5$. The active compound proportion prior hyperparameters were fixed as $a_\pi = b_\pi = 0.5 \sum_m n_m$. The hyperparameters specifying the number of plate specific clusters and global components were fixed as $H = K = 10$. The hyperparameters $\{\mu_{10}, \mu_{00}, a, b\}$ are computed based on the compound data and via equations (13) and (14), as described in the main paper.

D Model Convergence and Mixing

To assess convergence and mixing of the Gibbs sampler, we follow Neal [2], and provide trace and autocorrelation function (ACF) plots of some latent variables. We performed a total of 7000 iterations, discarding the first 3500 samples and using the remaining 3500 samples. We show plots of the global parameters μ_{1k}, μ_{0k}, σ_{1k}^2, σ_{0k}^2, and the global mixing components $\lambda_k^{(1)}$ and $\lambda_k^{(0)}$, for each of the synthetically generated data sets. At each iteration of the sampler, the respective parameters were sorted in increasing order to avoid label switching. The trace and ACF plots show good mixing rates and hence efficiency of the Bayesian computational algorithm.

[1] Subscript mi spans over plates and compounds (hence J_{mi} is a compound indicator), while subscript mh spans over plates and clusters (hence J_{mh} is a cluster indicator).

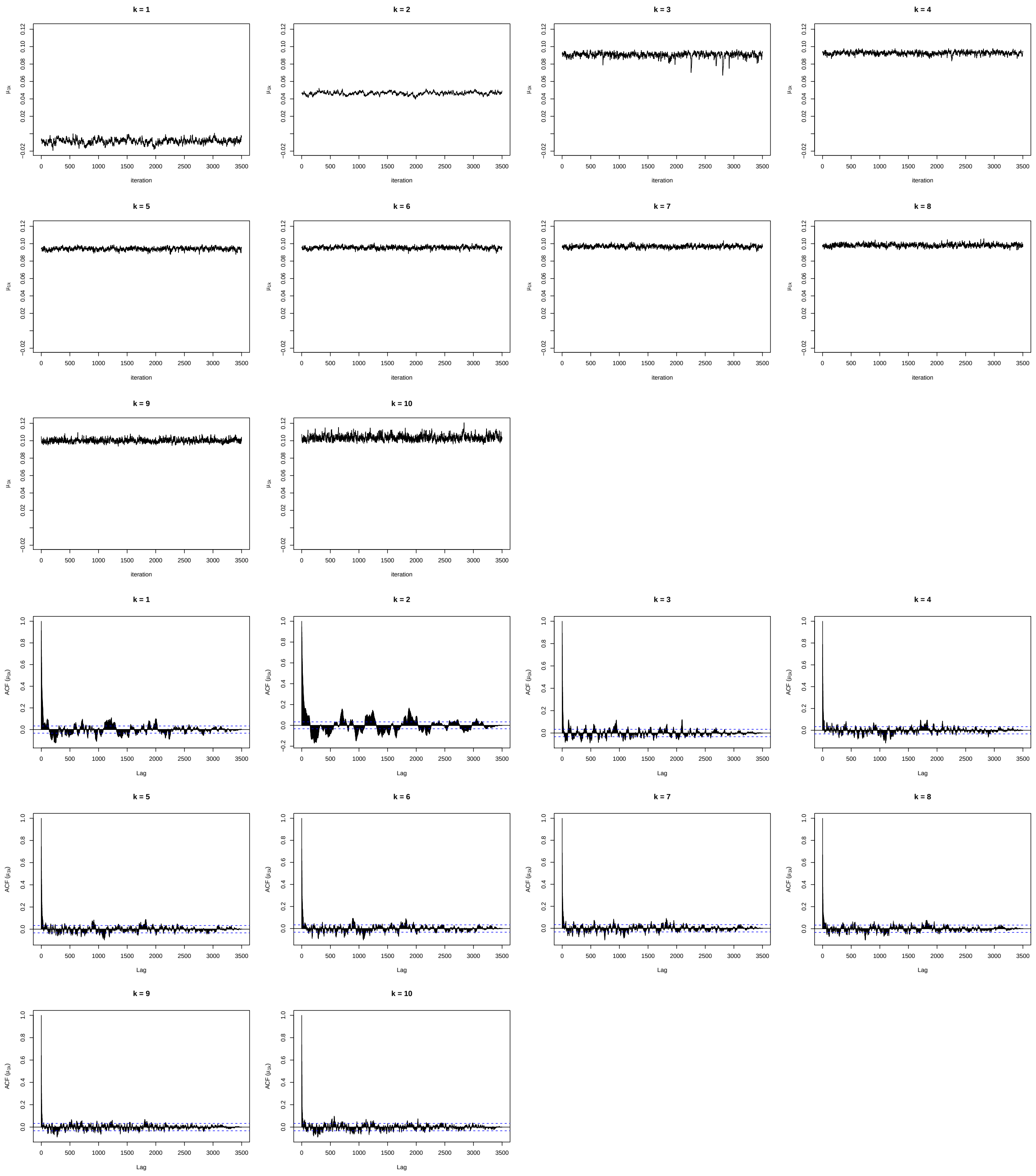

Figure 1: Trace and ACF plots of μ_{1k}. Synthetic Data Containing 10% Active Compounds.

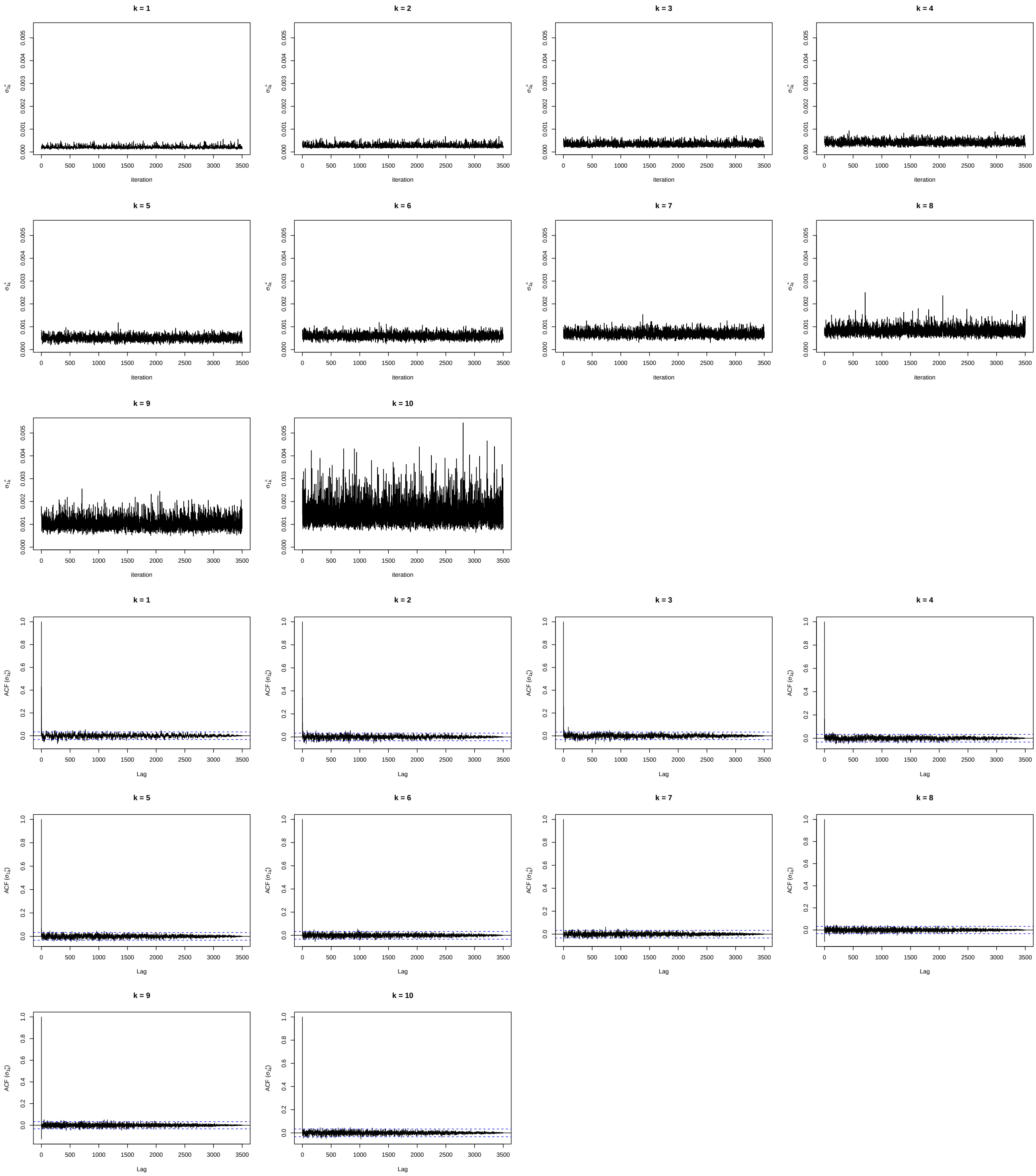

Figure 2: Trace and ACF plots of σ_{1k}^2. Synthetic Data Containing 10% Active Compounds.

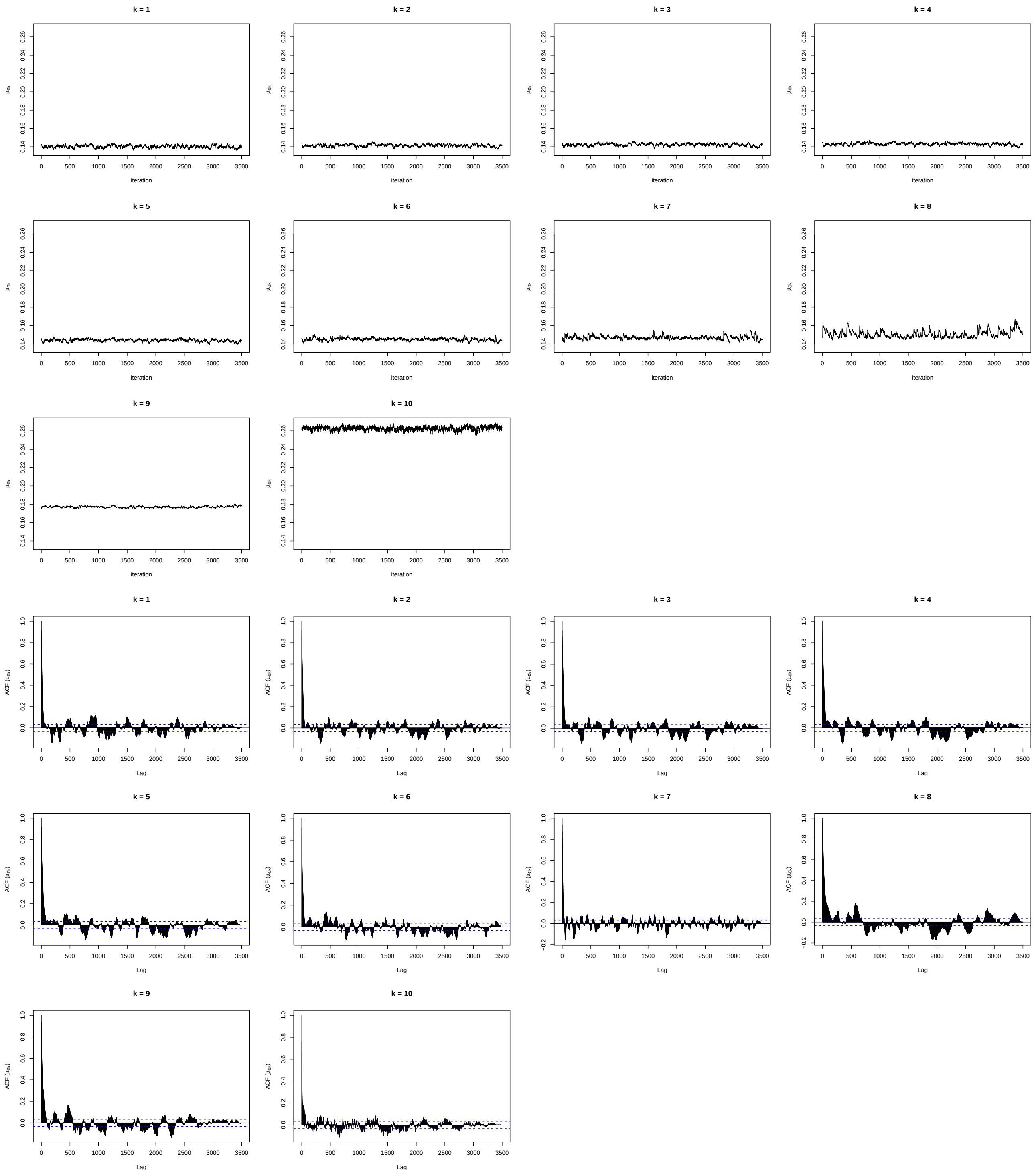

Figure 3: Trace and ACF plots of μ_{0k}. Synthetic Data Containing 10% Active Compounds.

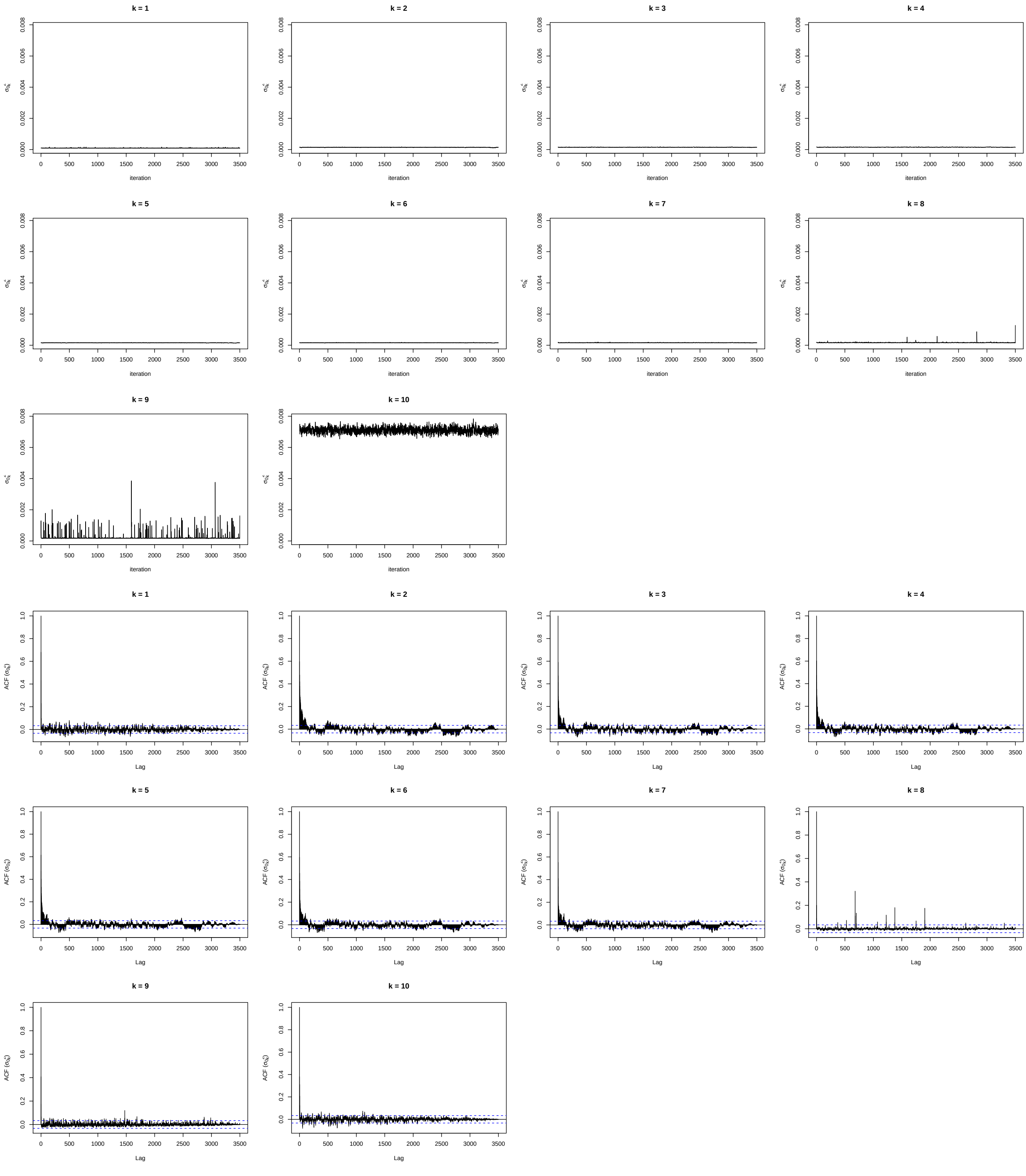

Figure 4: Trace and ACF plots of σ^2_{0k}. Synthetic Data Containing 10% Active Compounds.

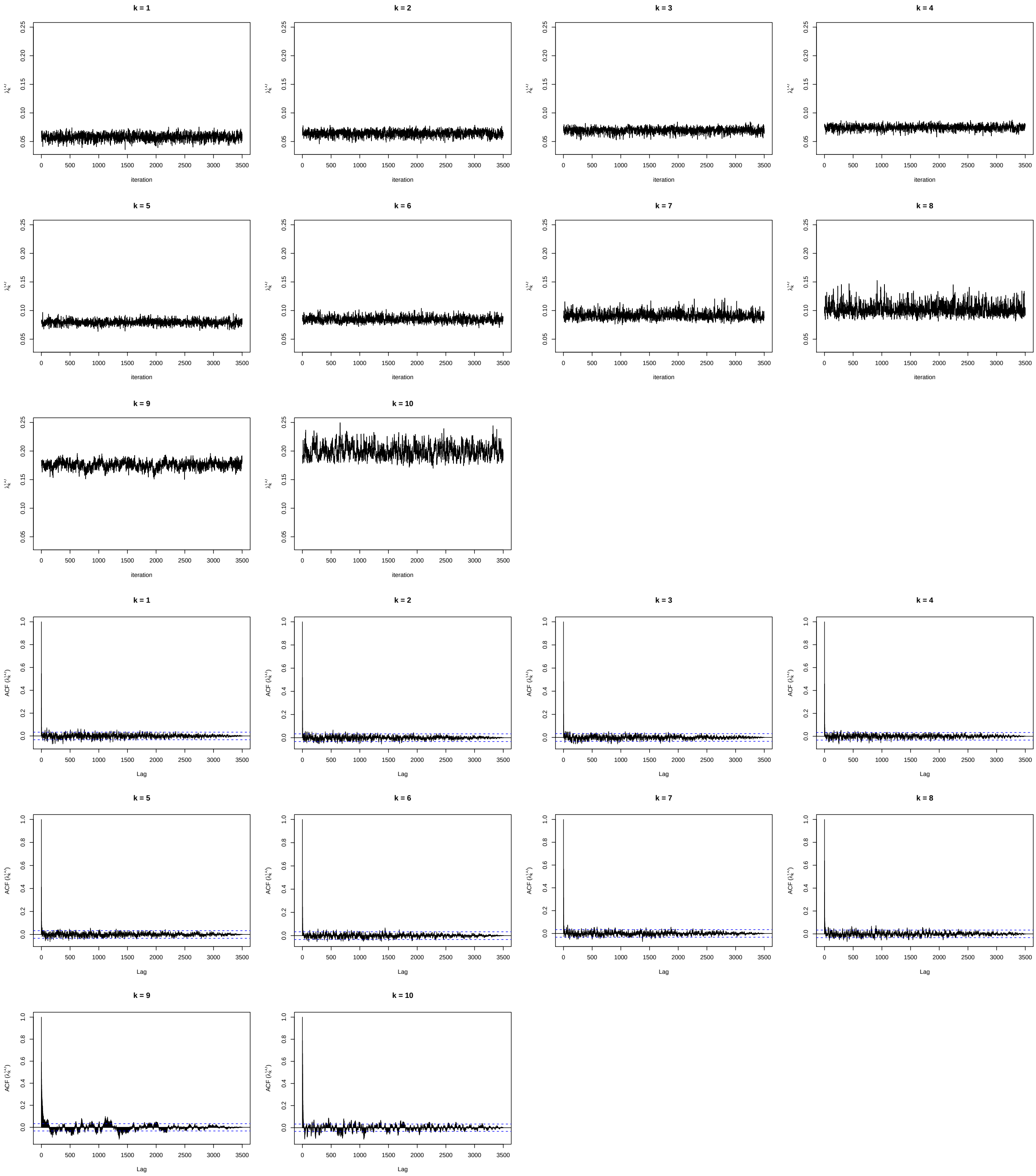

Figure 5: Trace and ACF plots of $\lambda_k^{(1)}$. Synthetic Data Containing 10% Active Compounds.

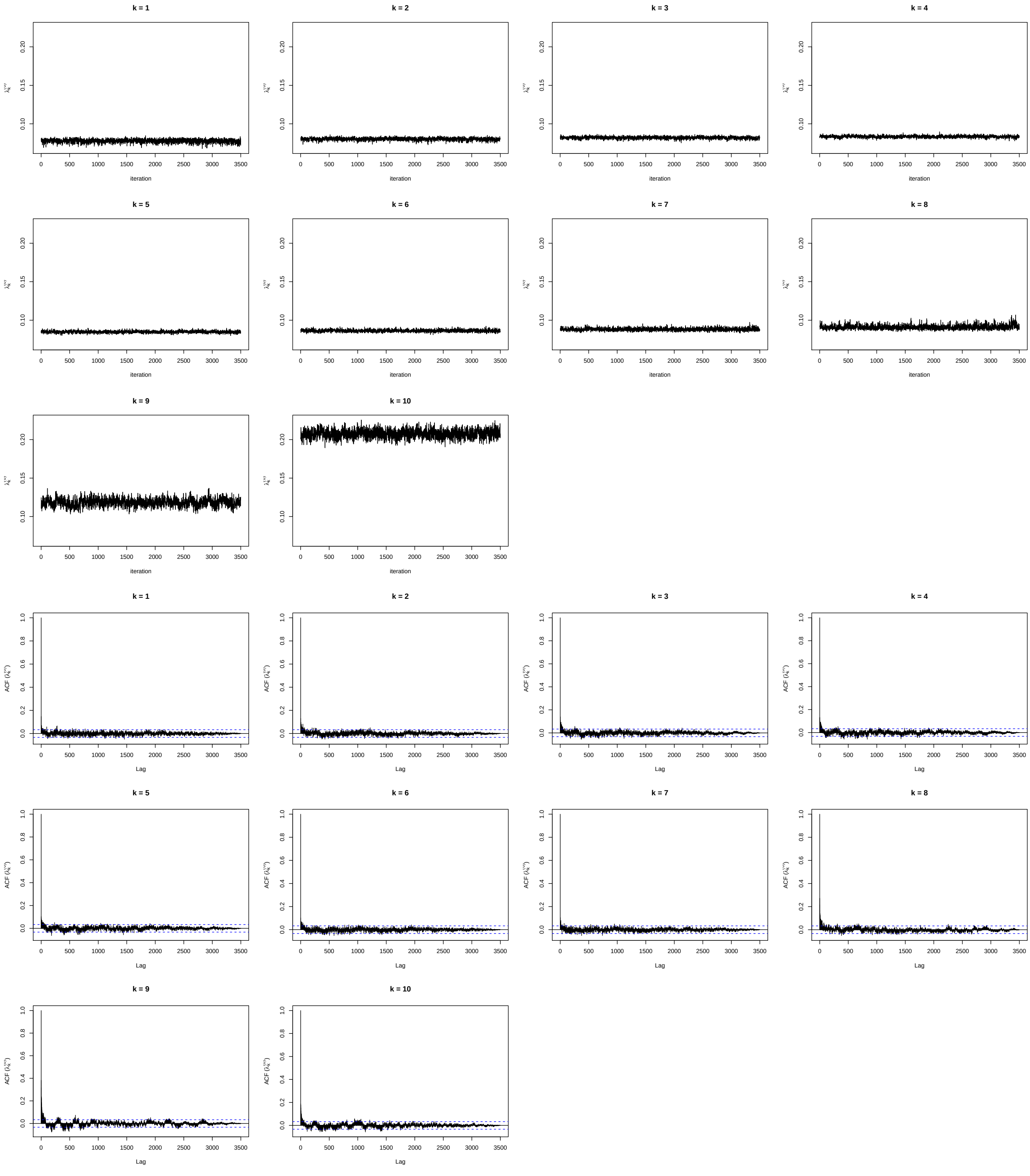

Figure 6: Trace and ACF plots of $\lambda_k^{(0)}$. Synthetic Data Containing 10% Active Compounds.

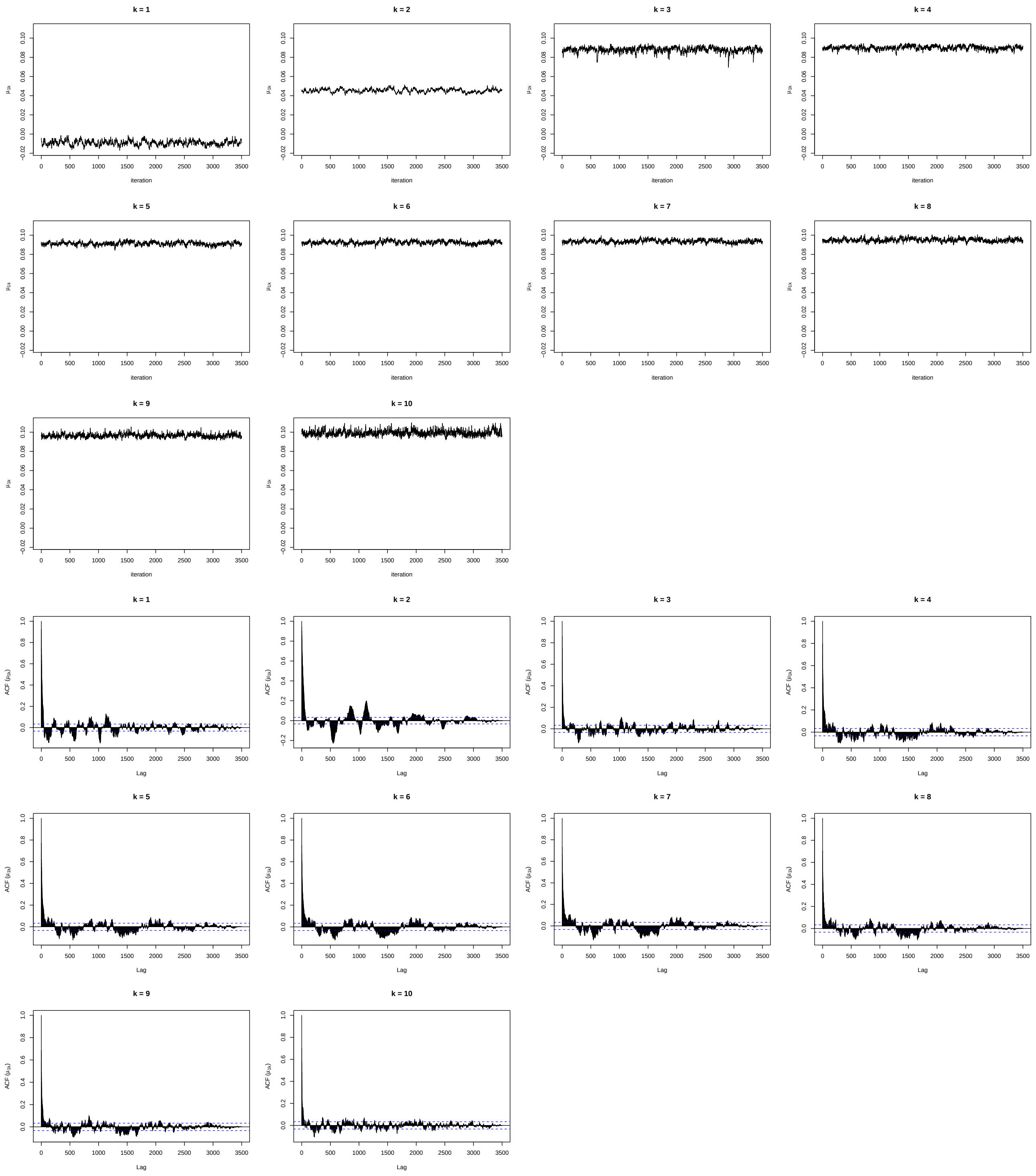

Figure 7: Trace and ACF plots of μ_{1k}. Synthetic Data Containing 5% Active Compounds.

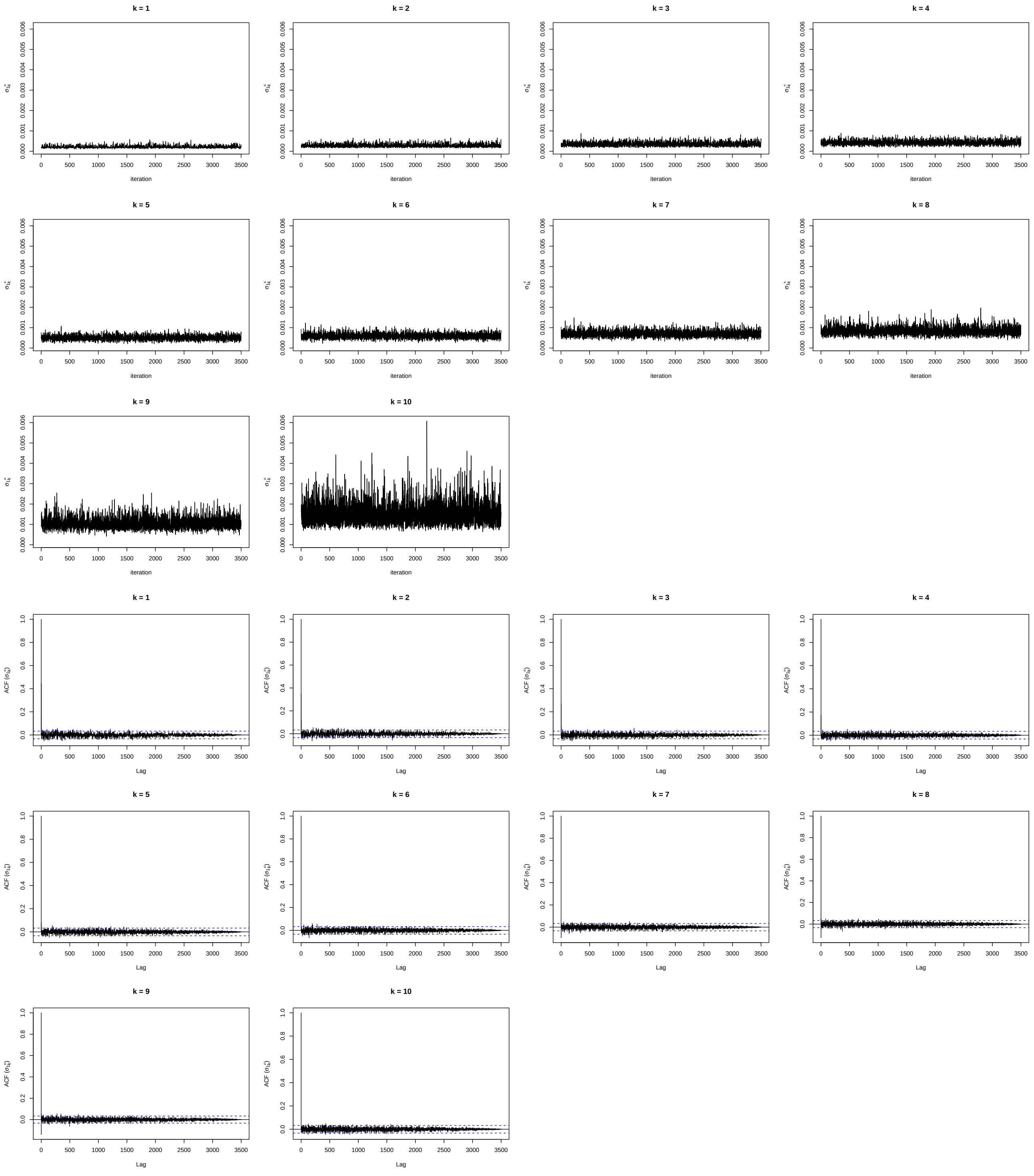

Figure 8: Trace and ACF plots of σ^2_{1k}. Synthetic Data Containing 5% Active Compounds.

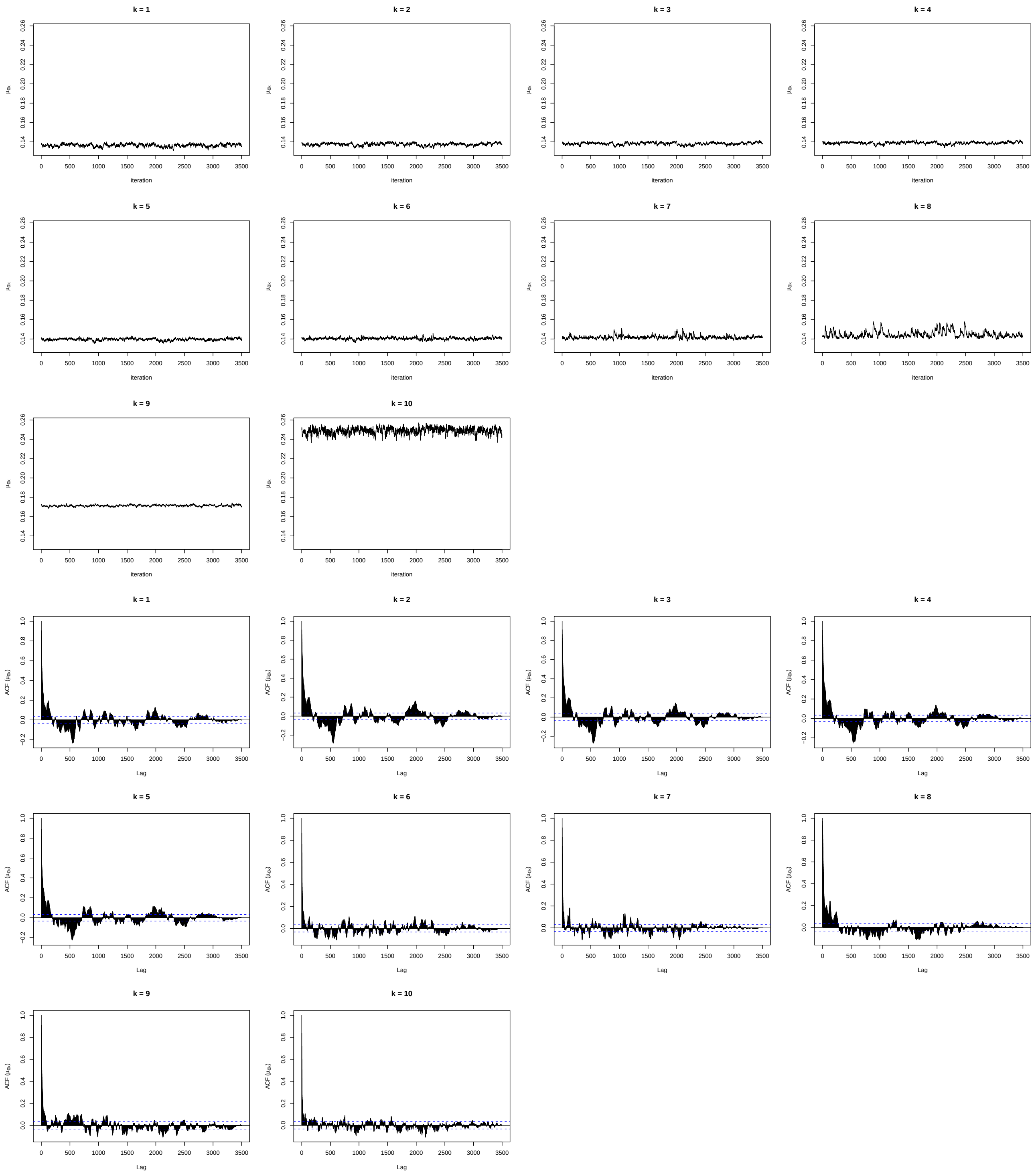

Figure 9: Trace and ACF plots of μ_{0k}. Synthetic Data Containing 5% Active Compounds.

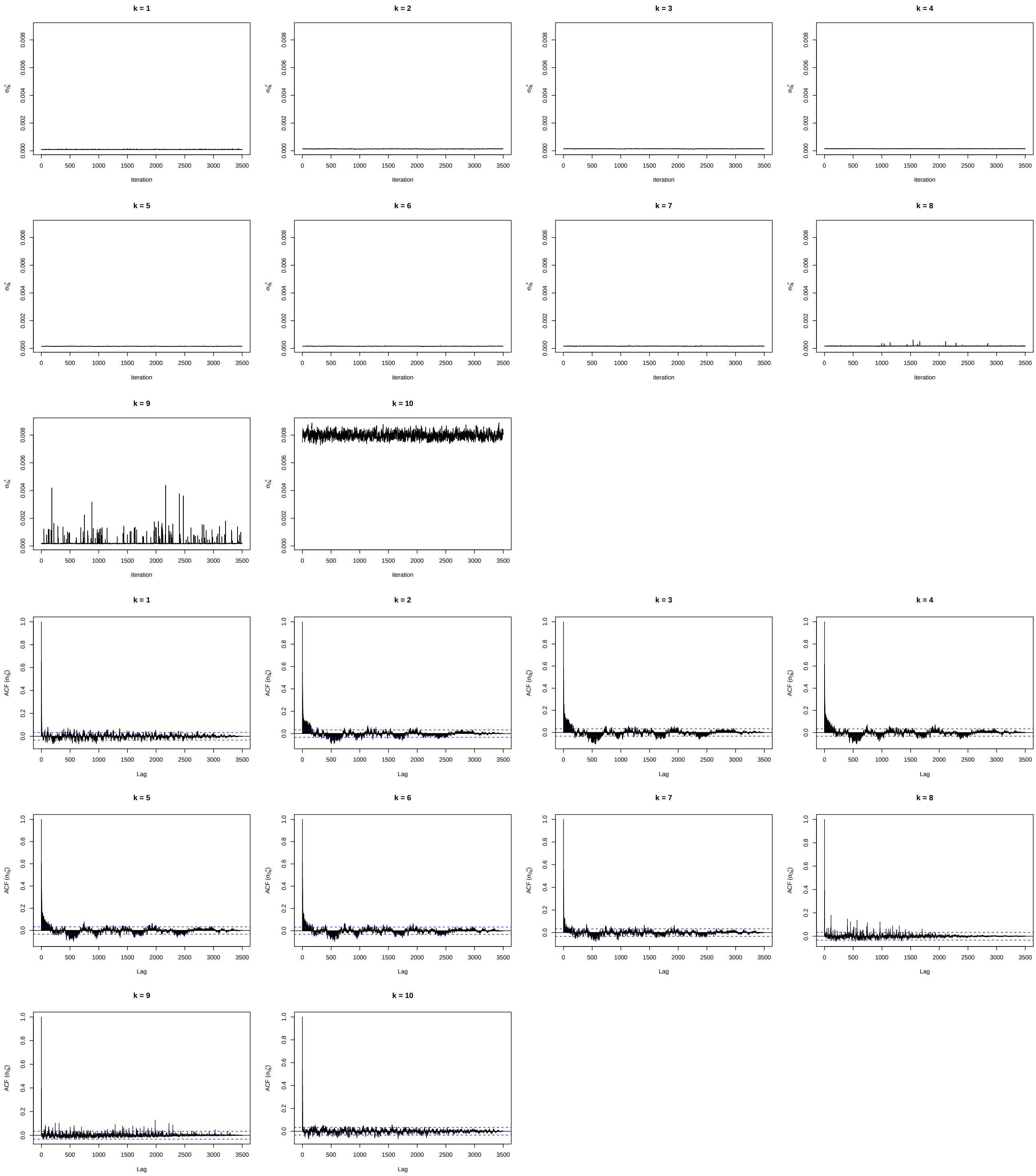

Figure 10: Trace and ACF plots of σ^2_{0k}. Synthetic Data Containing 5% Active Compounds.

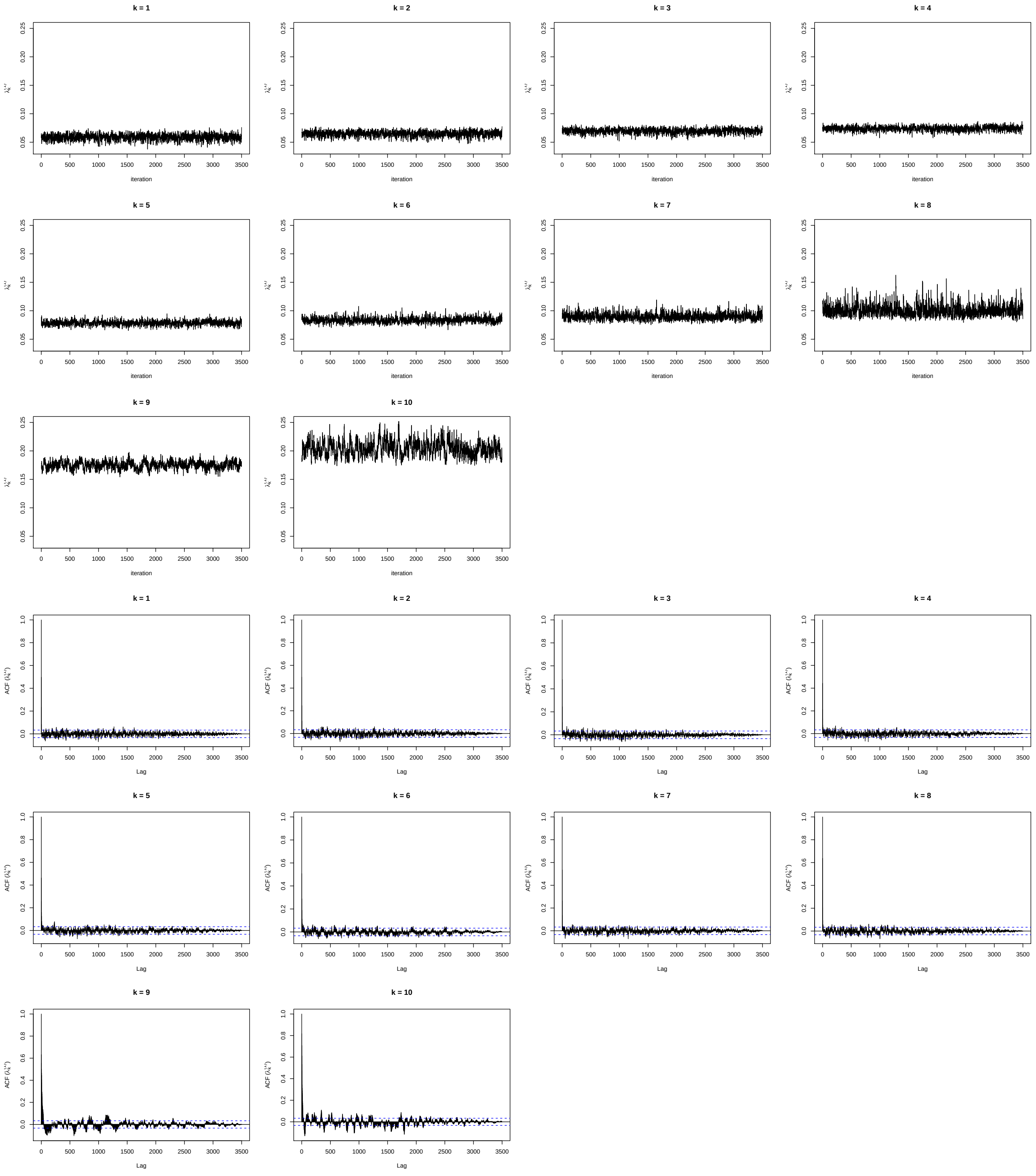

Figure 11: Trace and ACF plots of $\lambda_k^{(1)}$. Synthetic Data Containing 5% Active Compounds.

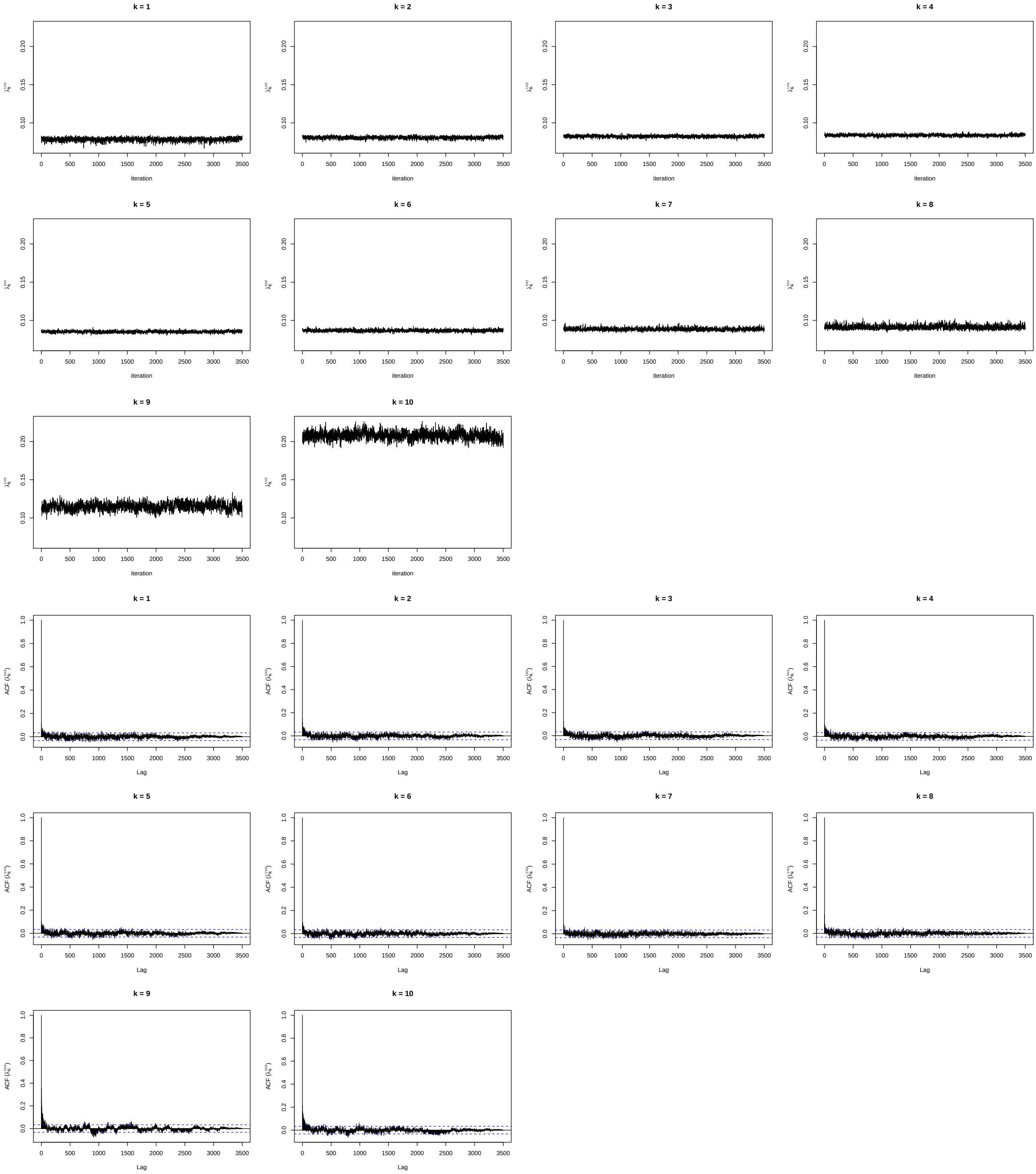

Figure 12: Trace and ACF plots of $\lambda_k^{(0)}$. Synthetic Data Containing 5% Active Compounds.

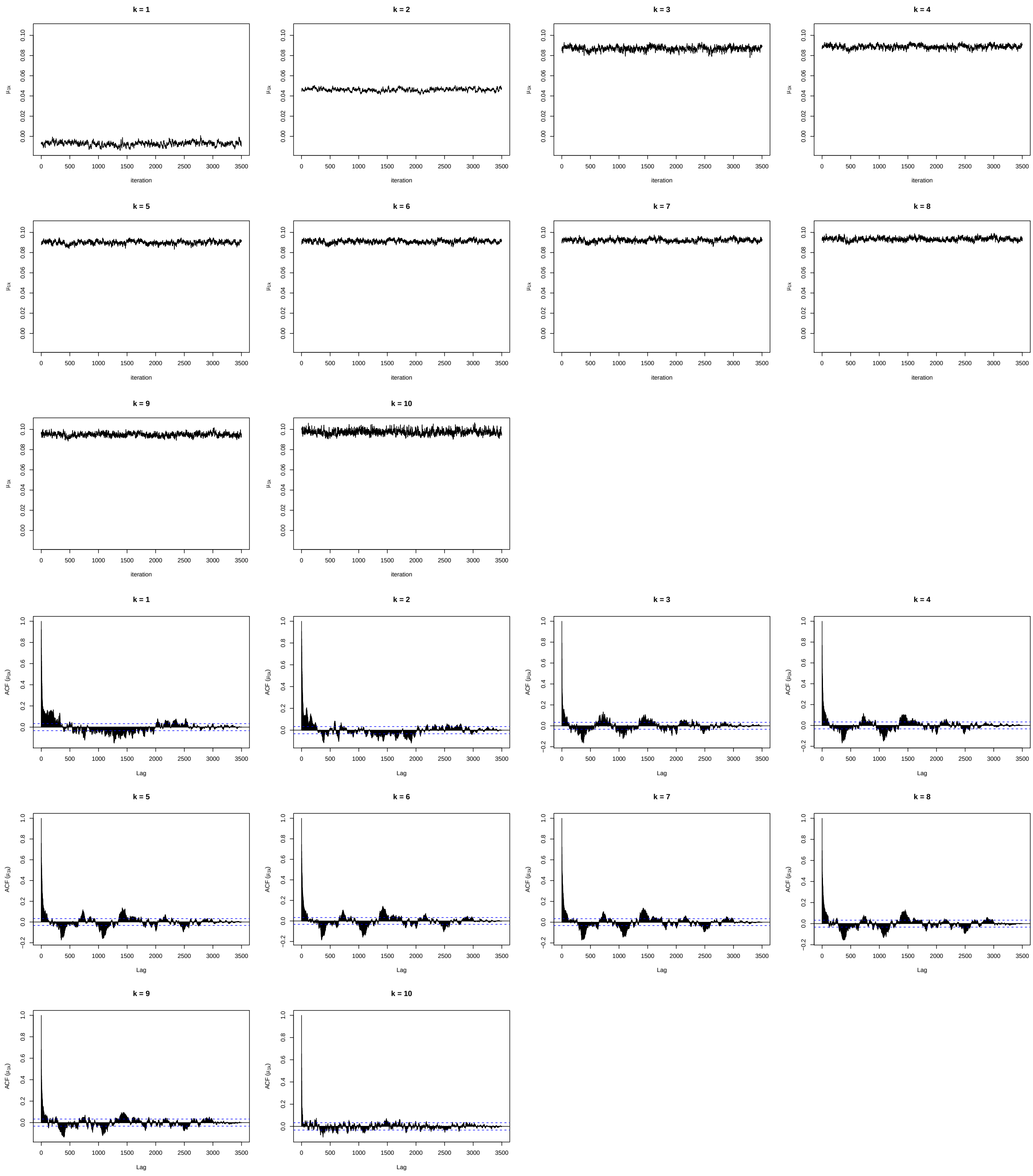

Figure 13: Trace and ACF plots of μ_{1k}. Synthetic Data Containing 1% Active Compounds.

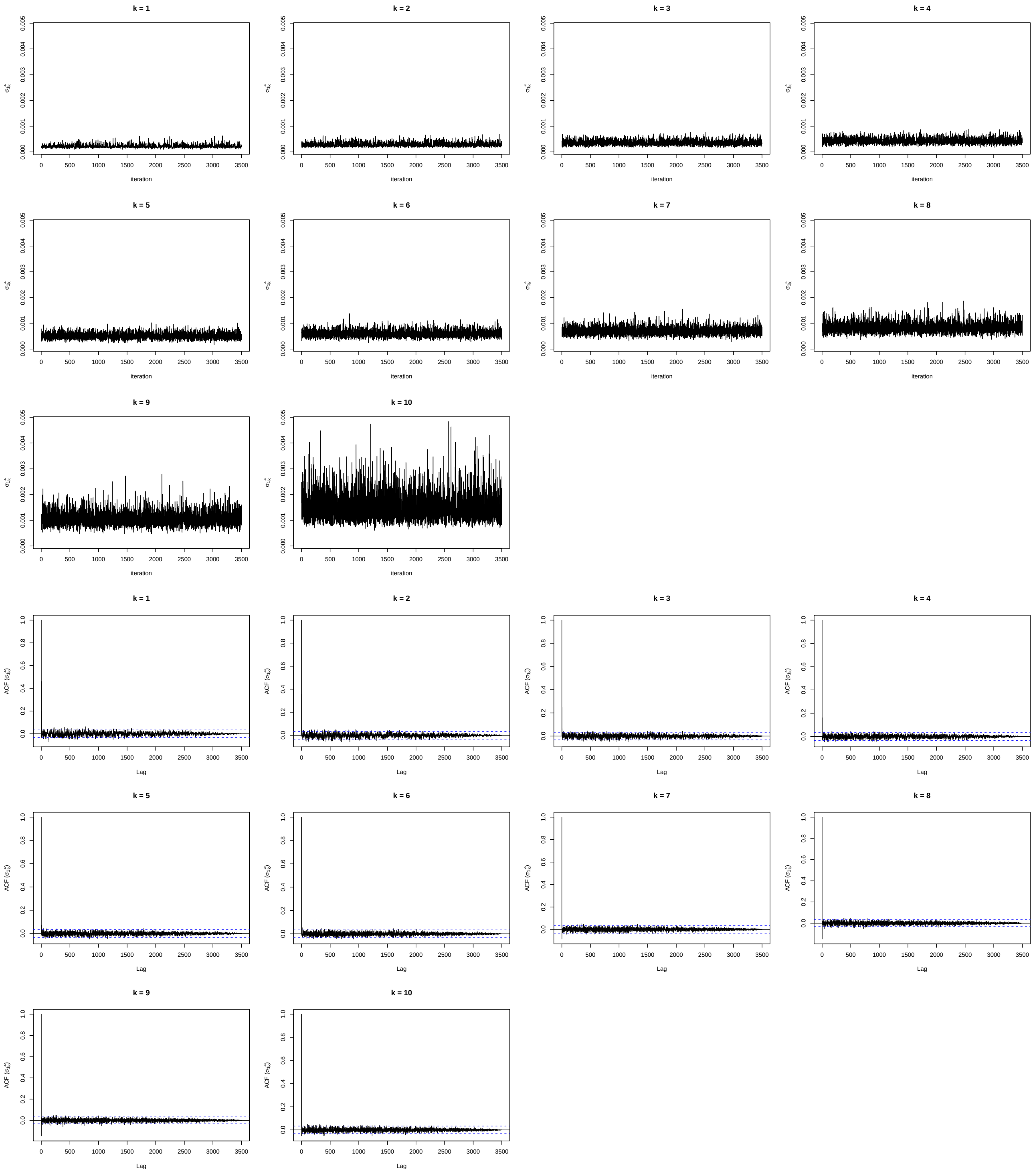

Figure 14: Trace and ACF plots of σ^2_{1k}. Synthetic Data Containing 1% Active Compounds.

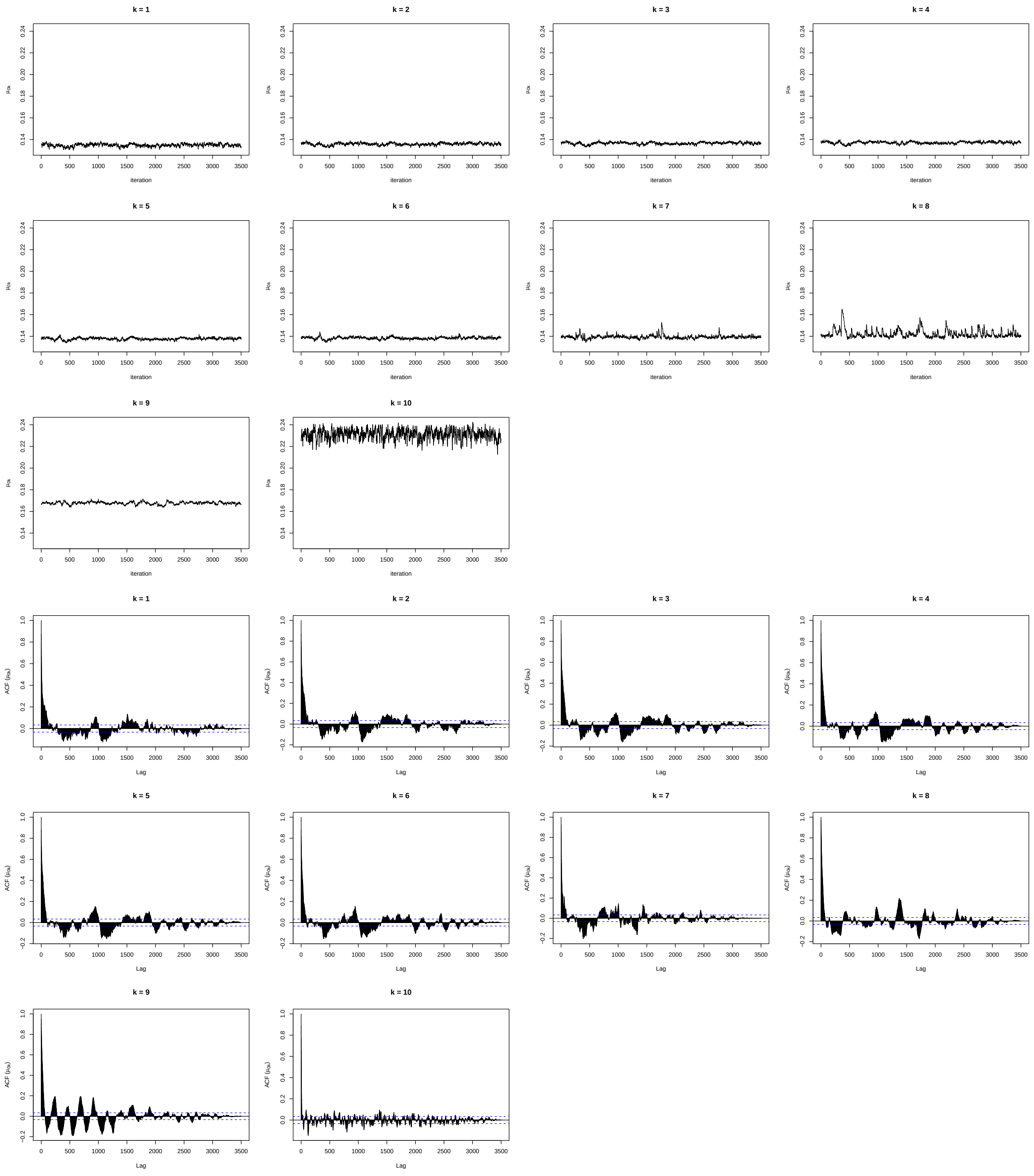

Figure 15: Trace and ACF plots of μ_{0k}. Synthetic Data Containing 1% Active Compounds.

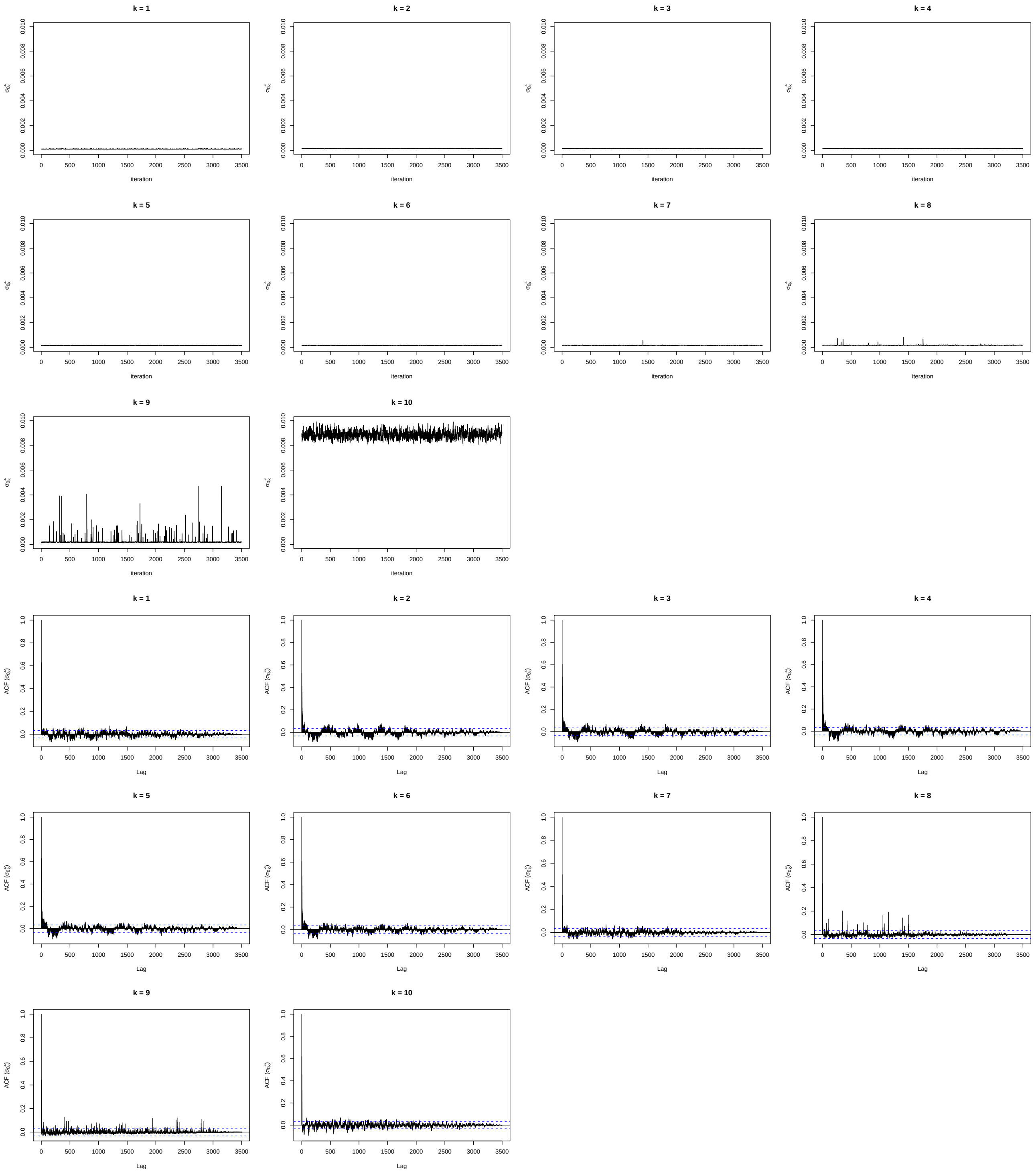

Figure 16: Trace and ACF plots of σ_{0k}^2. Synthetic Data Containing 1% Active Compounds.

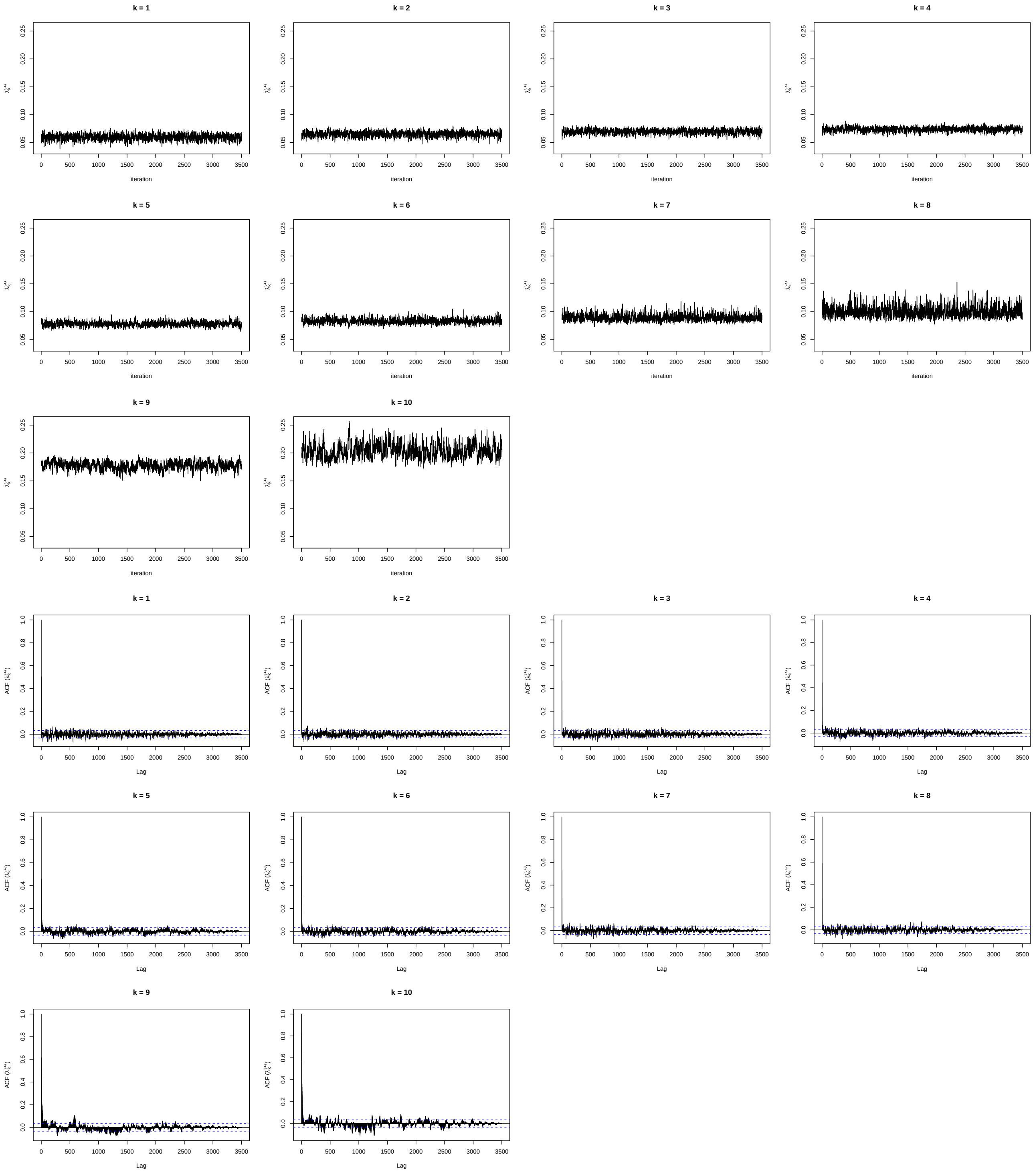

Figure 17: Trace and ACF plots of $\lambda_k^{(1)}$. Synthetic Data Containing 1% Active Compounds.

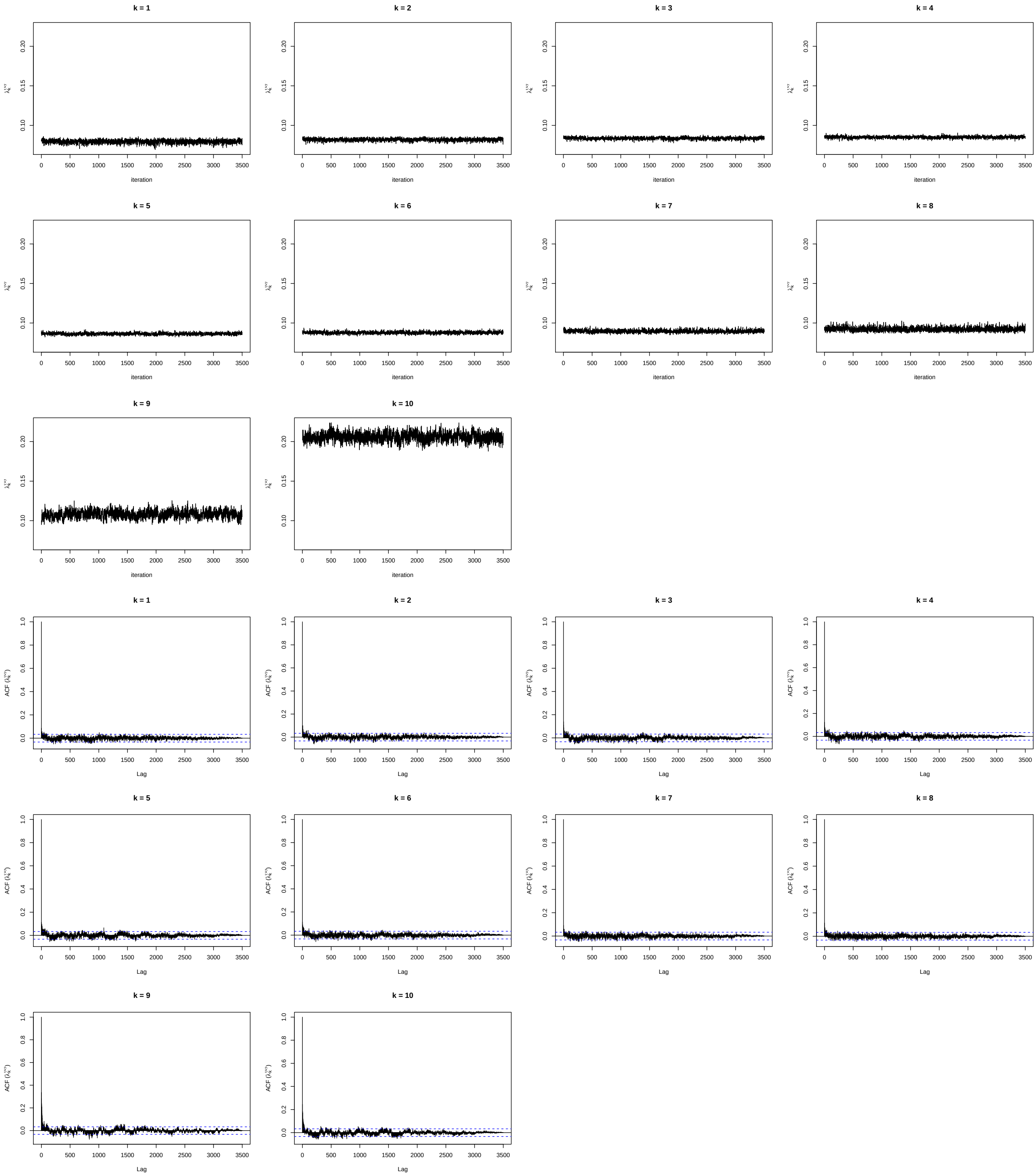

Figure 18: Trace and ACF plots of $\lambda_k^{(0)}$. Synthetic Data Containing 1% Active Compounds.

E Experiments with 384-Well Plates

In this section we carry out experiments with synthetic data, designed based on the 384-well plate format.

E.1 Synthetic Data Design

Following the procedure described in the main body of the paper, we generated a set of 320×10^3 compounds, randomly distributed in 1000 plates, consisting of hits and non-hits. In simulating plate effects, we generated random noise from the matrix-normal distribution, with a zero location matrix and row and column scale matrices shown in Fig. 19. Again, we used a real data set of compounds exhibiting the plate design shown in Fig. 2 of the main paper. Excluding the control well columns, we computed B-scores based on individual 16×20 compound well plates. The row-wise and column-wise covariance matrices were estimated as the difference between the compound raw values and their B-scores. The estimated covariance matrices were used (after proper scaling) as row and column scale matrices in generating the plate noise effects. To simulate plate areas of increased and decreased readouts, two randomly chosen 4×4 patches of wells per noise plate were multiplied by 1.5 and by 0.5, respectively. Slope effects were simulated by multiplying the plate columns by $\{1.01, 1.02, 1.03, 1.04, 1.05, 1.06, 1.07, 1.08, 1.09, 1.10, 1.11, 1.12, 1.13, 1.14, 1.15, 1.16, 1.17, 1.18, 1.19, 1.20\}$, respectively. An independently drawn noise plate (examples shown in Fig. 20) was added to each of the compound plates. The resulting data plates were used as test data.

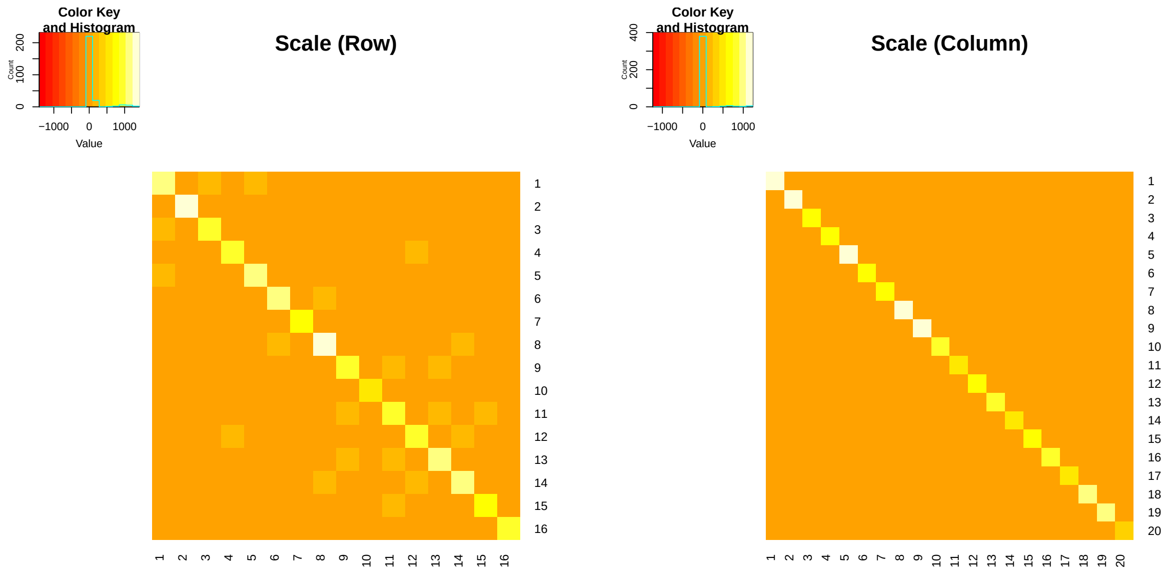

Figure 19: Scale matrices used in generating the synthetic noise plates, indicating predominantly row dependent within plate effects.

E.2 Choice of Hyperparameters

Prior to applying the BHTS, B-score and R-score methods, we normalized the data so that each plate had a mean zero and variance one. We found that this normalization can improve mixing of the sampler.

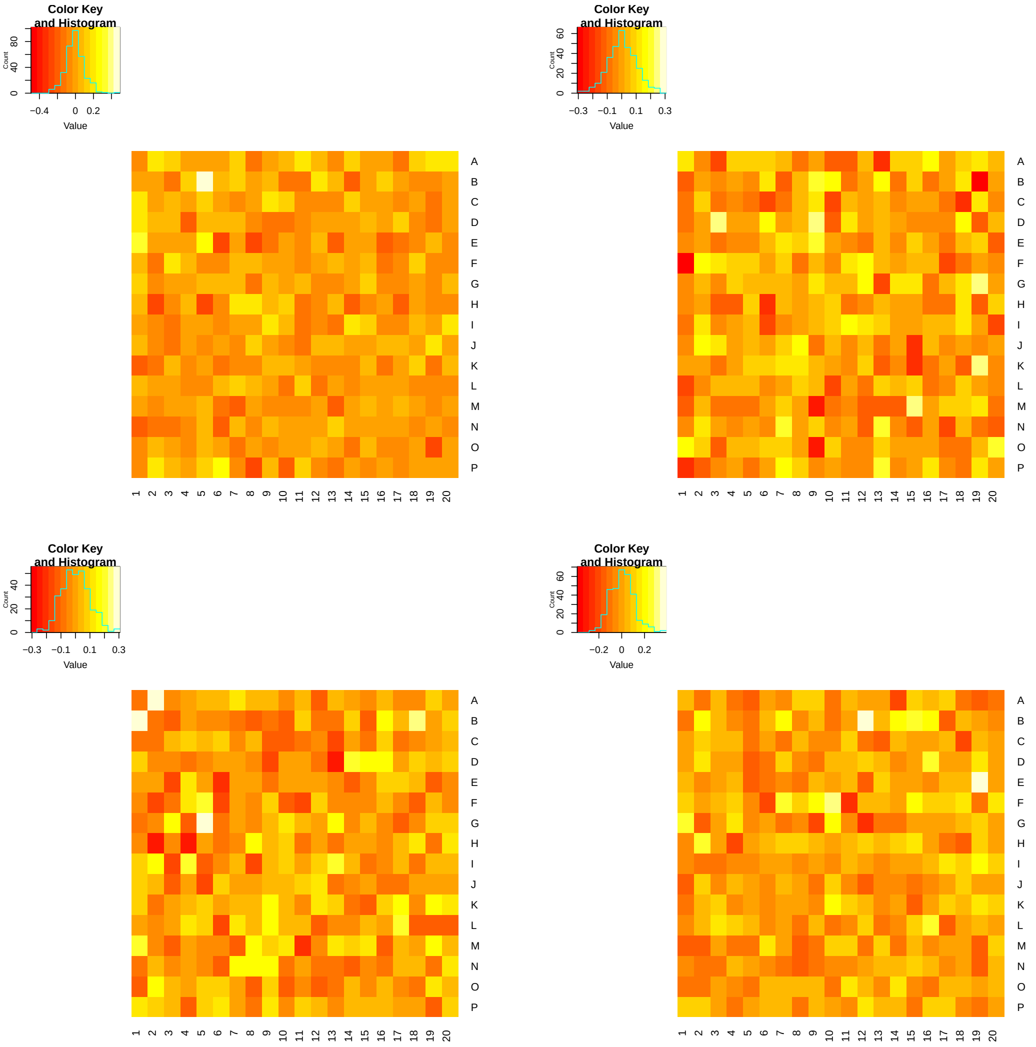

Figure 20: Examples of noise plates.

The hyperparameters $\{a_\alpha, b_\alpha, a_\tau, b_\tau, a_\pi, b_\pi, \mu_{10}, \mu_{00}, a, b\}$ are fixed in the same way as in the previous experiments. The hyperparameters specifying the number of plate specific clusters and global components were fixed as $H = 15$ and $K = 10$, respectively.

E.3 Comparison with B-score and R-score Methods

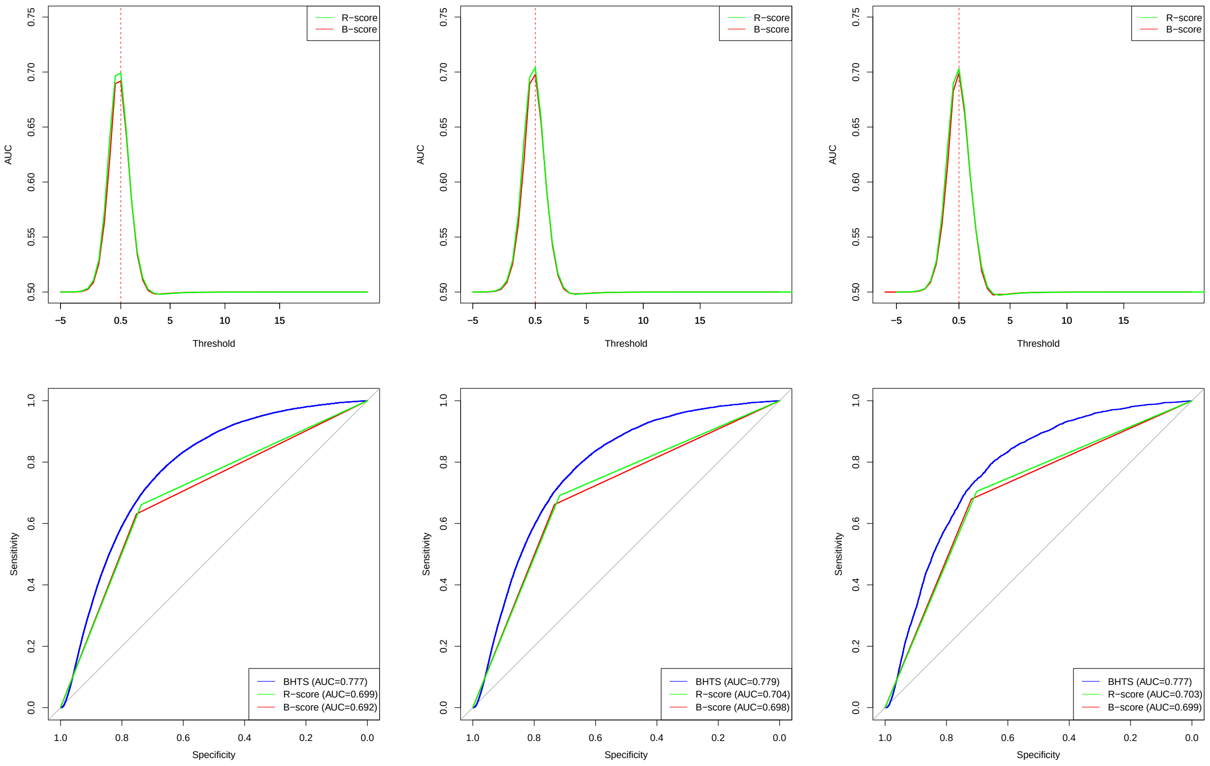

Figure 21: Top row shows AUC plots of B and R score methods, as functions of thresholds. Bottom row shows ROC plots of the B-score, R-score and BHTS methods. Data sets containing 10% (left column), 5% (middle column) and 1% (right column) of active compounds, respectively. The piecewise-linear shape of the B and R score curves is due to the binary nature of the predictor.

E.4 Model Convergence and Mixing

We performed a total of 10000 iterations, discarding the first 5000 samples and using the remaining 5000 samples (shown in the plots). We show trace and ACF plots of the global parameters μ_{1k}, μ_{0k}, σ^2_{1k}, σ^2_{0k}, and the global mixing components $\lambda^{(1)}_k$ and $\lambda^{(0)}_k$, for each of the synthetically generated data sets. At each iteration of the sampler, the respective parameters were sorted in increasing order to avoid label switching. The trace and ACF plots show good mixing rates and hence efficiency of the Bayesian computational algorithm.

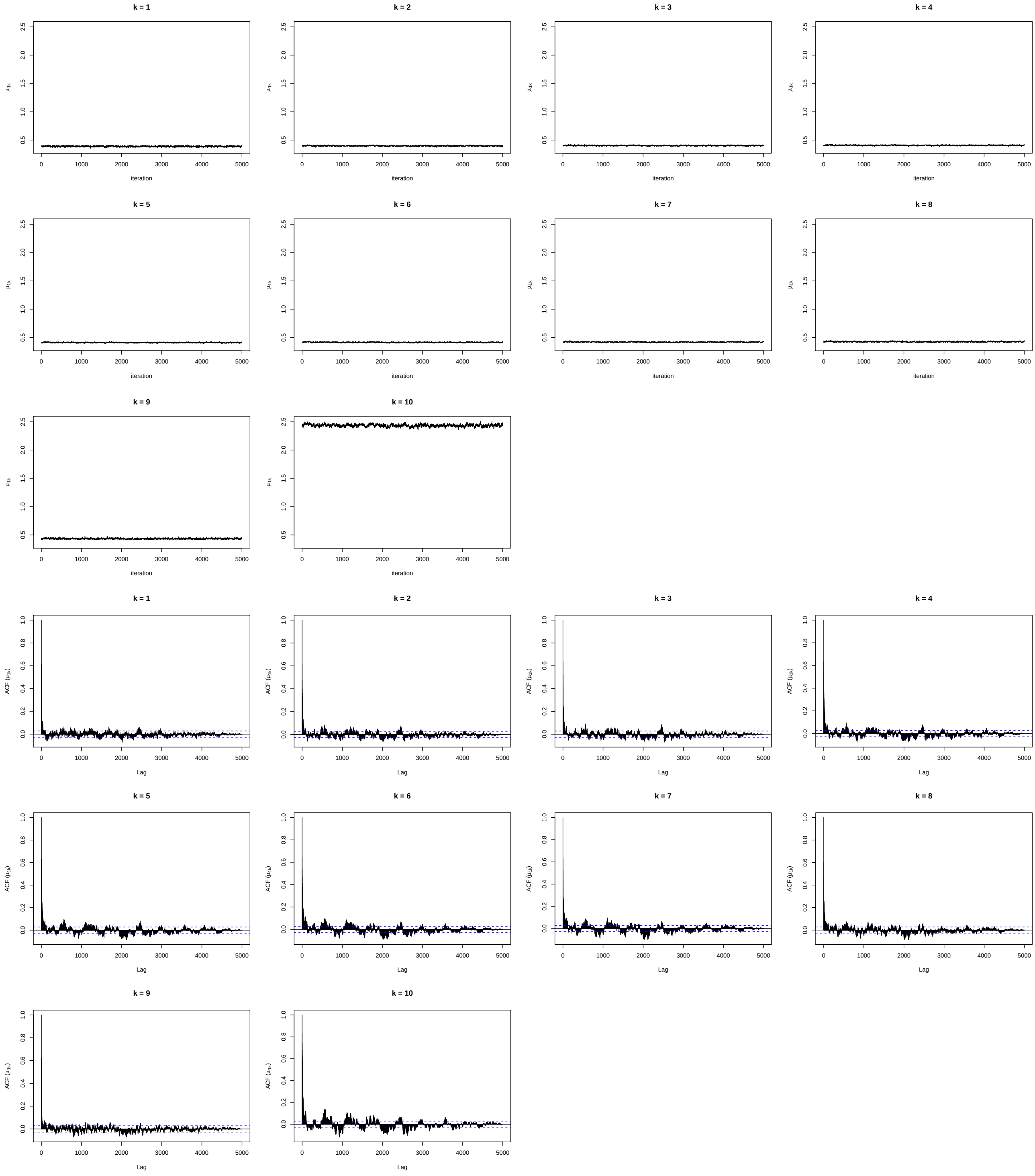

Figure 22: Trace and ACF plots of μ_{1k}. Synthetic Data Containing 10% Active Compounds.

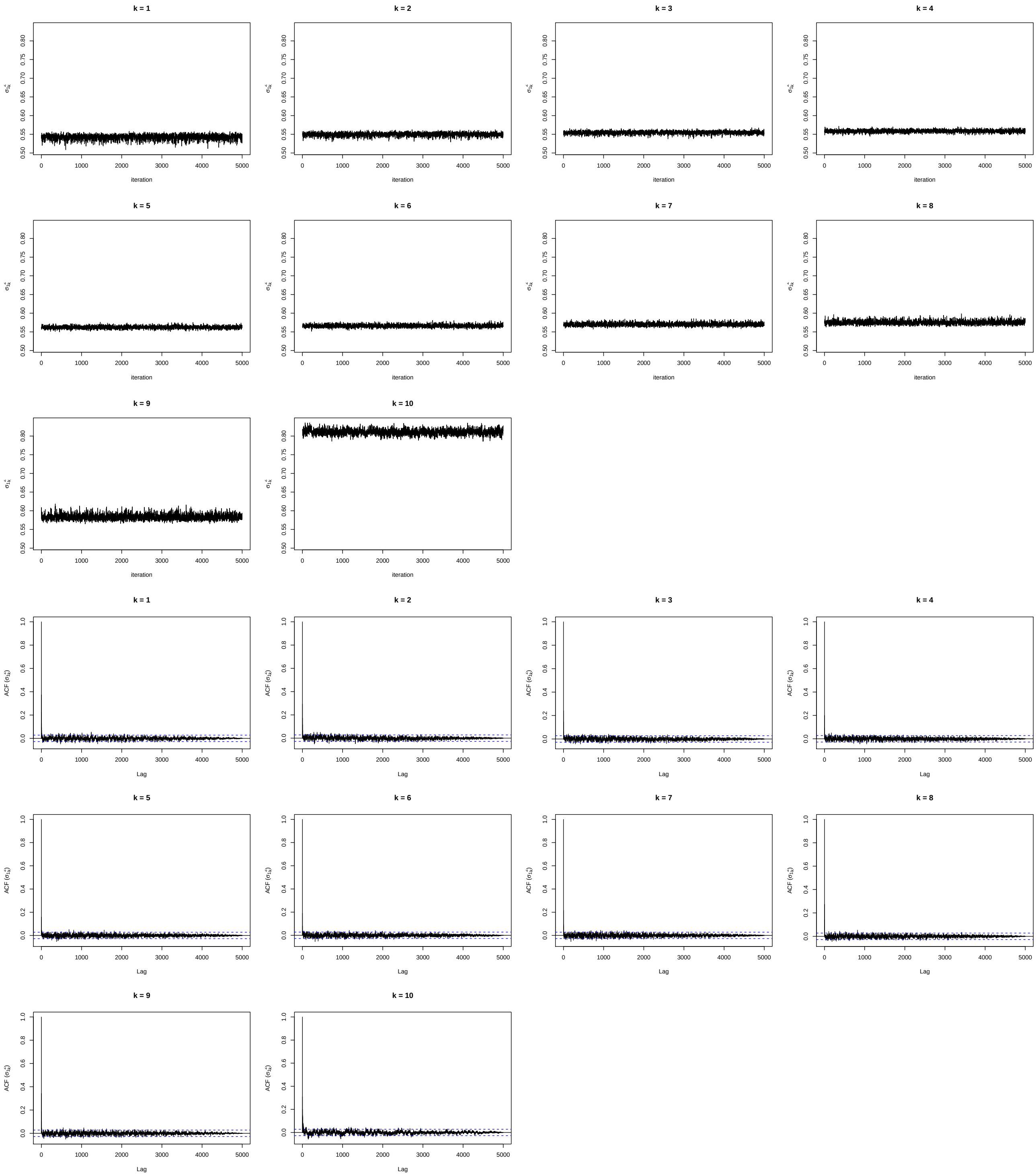

Figure 23: Trace and ACF plots of σ^2_{1k}. Synthetic Data Containing 10% Active Compounds.

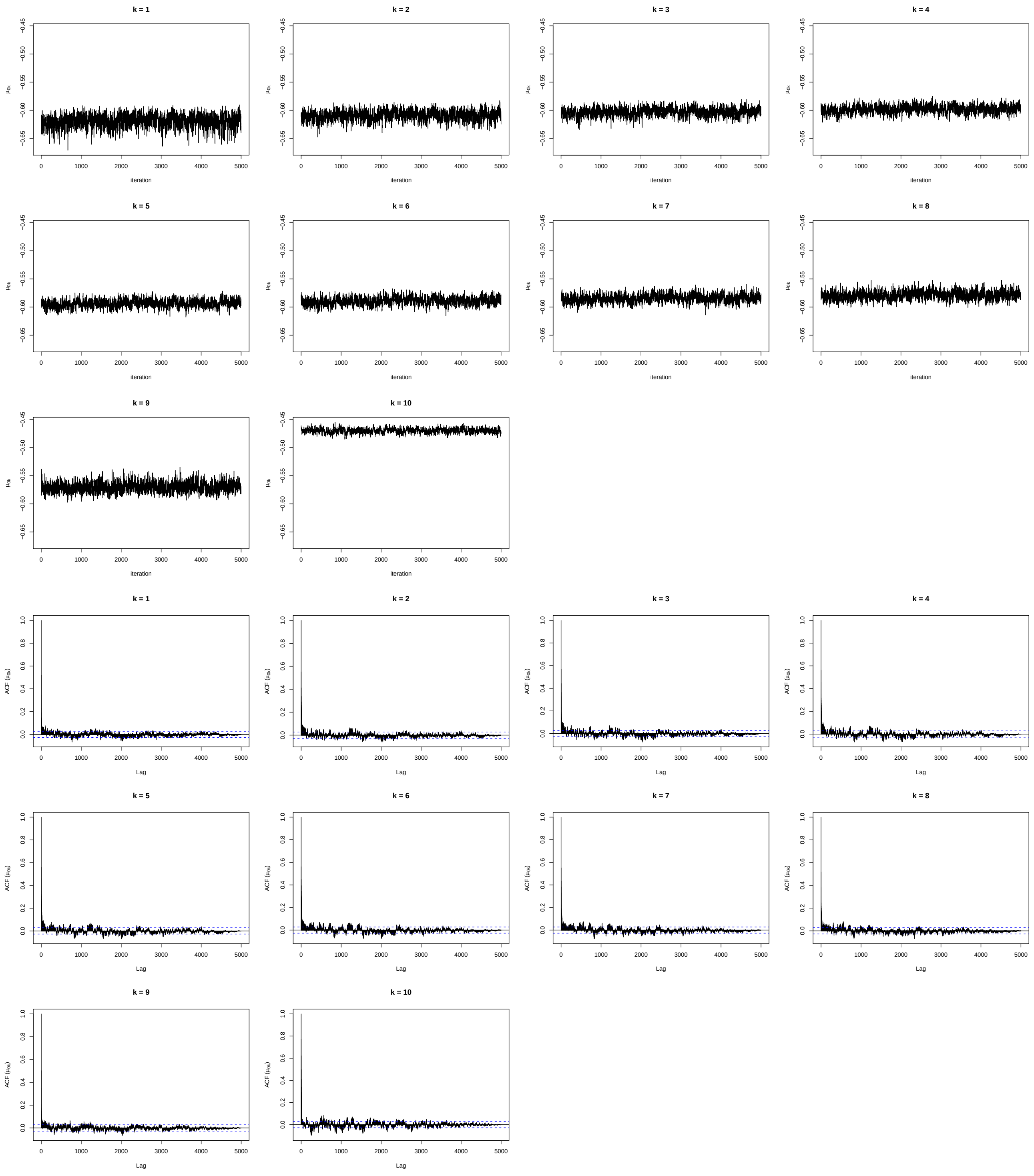

Figure 24: Trace and ACF plots of μ_{0k}. Synthetic Data Containing 10% Active Compounds.

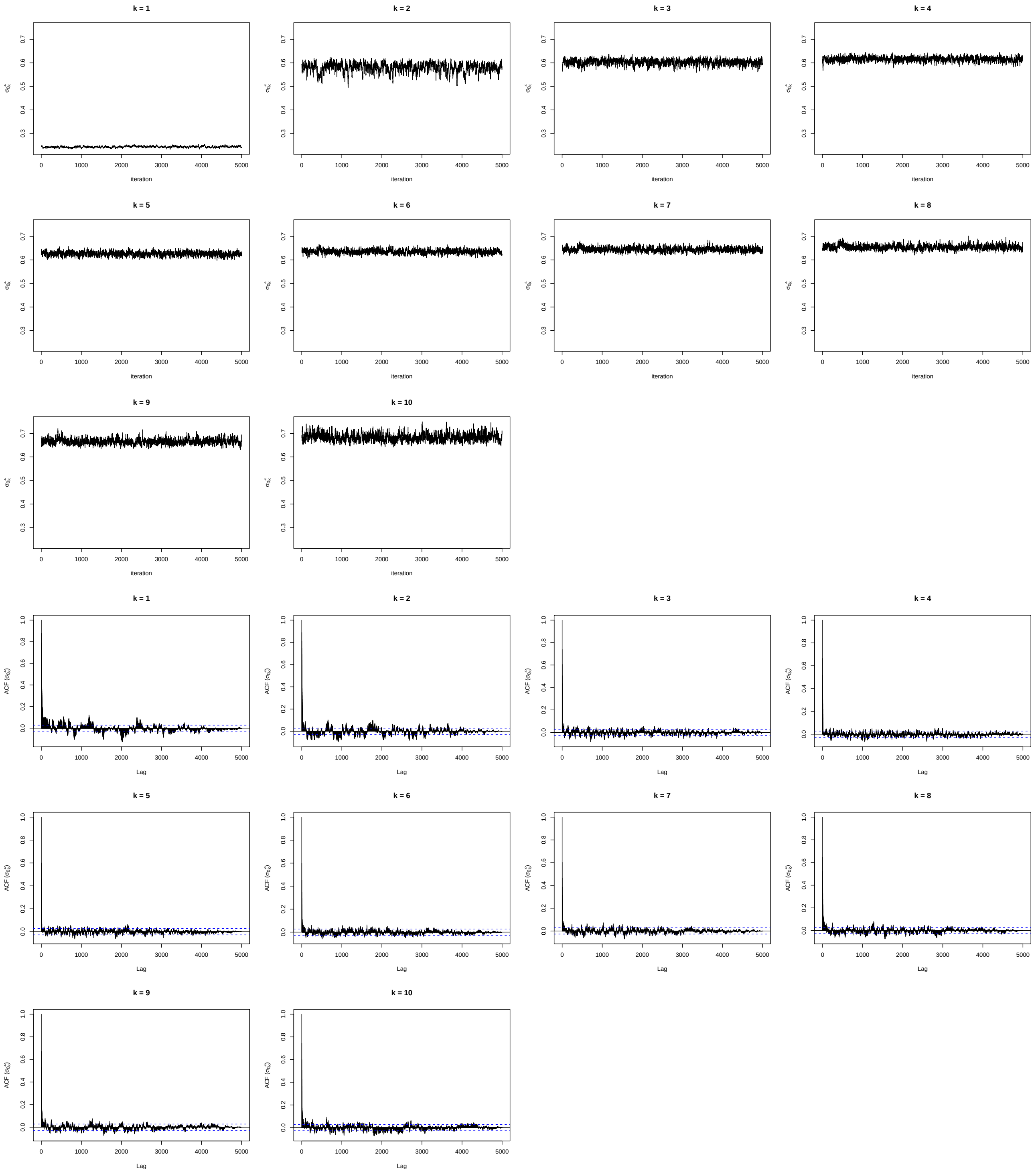

Figure 25: Trace and ACF plots of σ_{0k}^2. Synthetic Data Containing 10% Active Compounds.

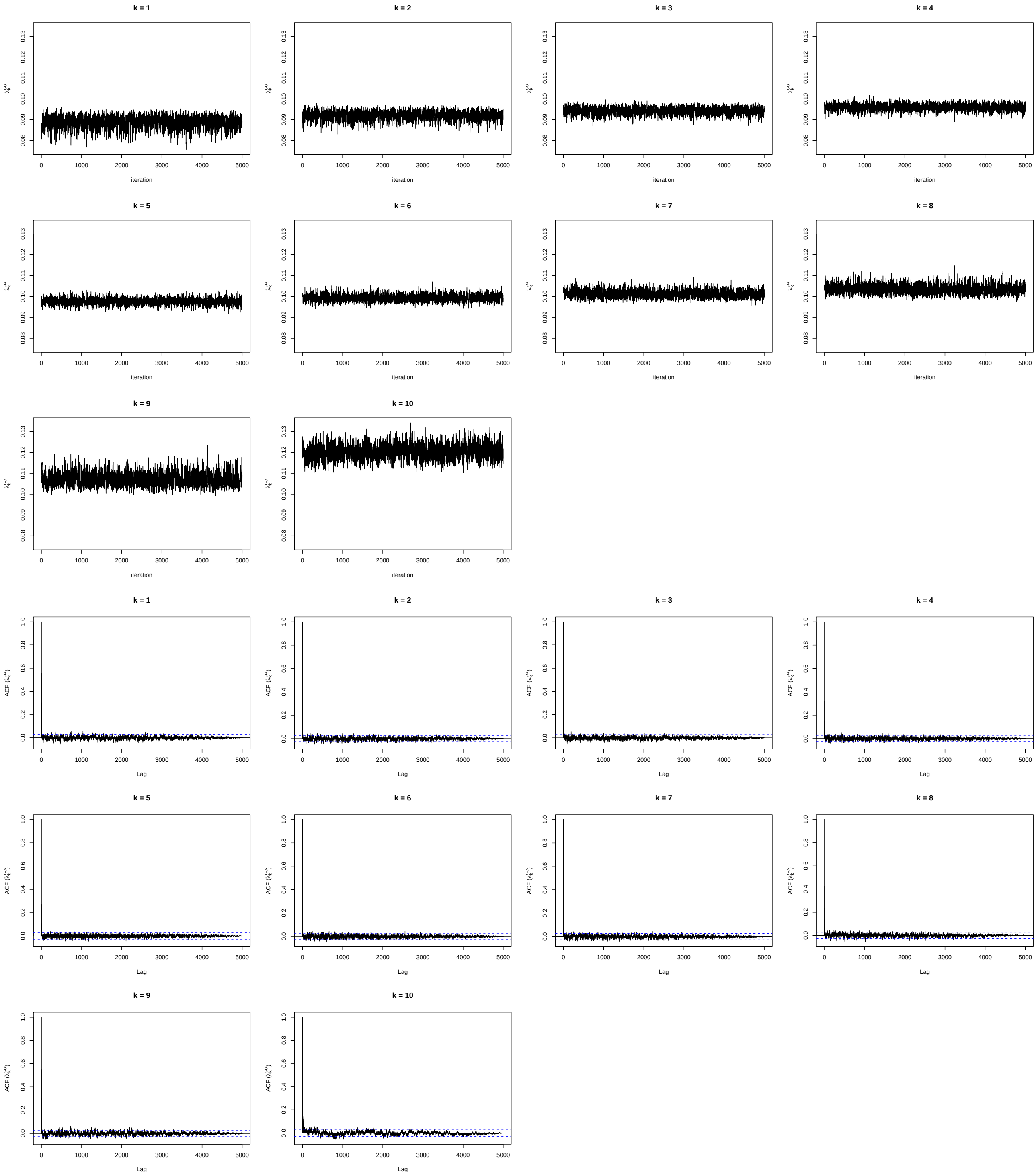

Figure 26: Trace and ACF plots of $\lambda_k^{(1)}$. Synthetic Data Containing 10% Active Compounds.

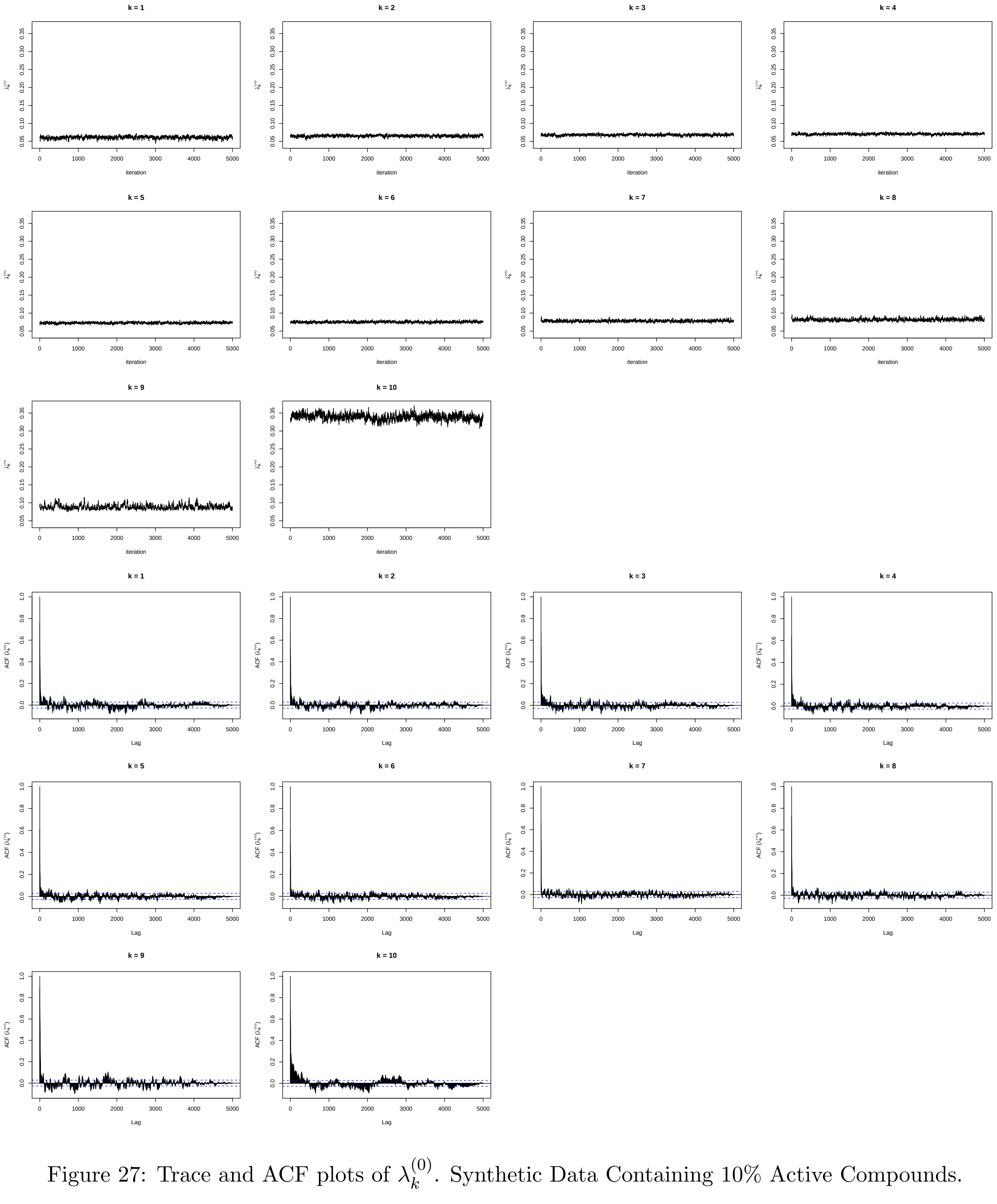

Figure 27: Trace and ACF plots of $\lambda_k^{(0)}$. Synthetic Data Containing 10% Active Compounds.

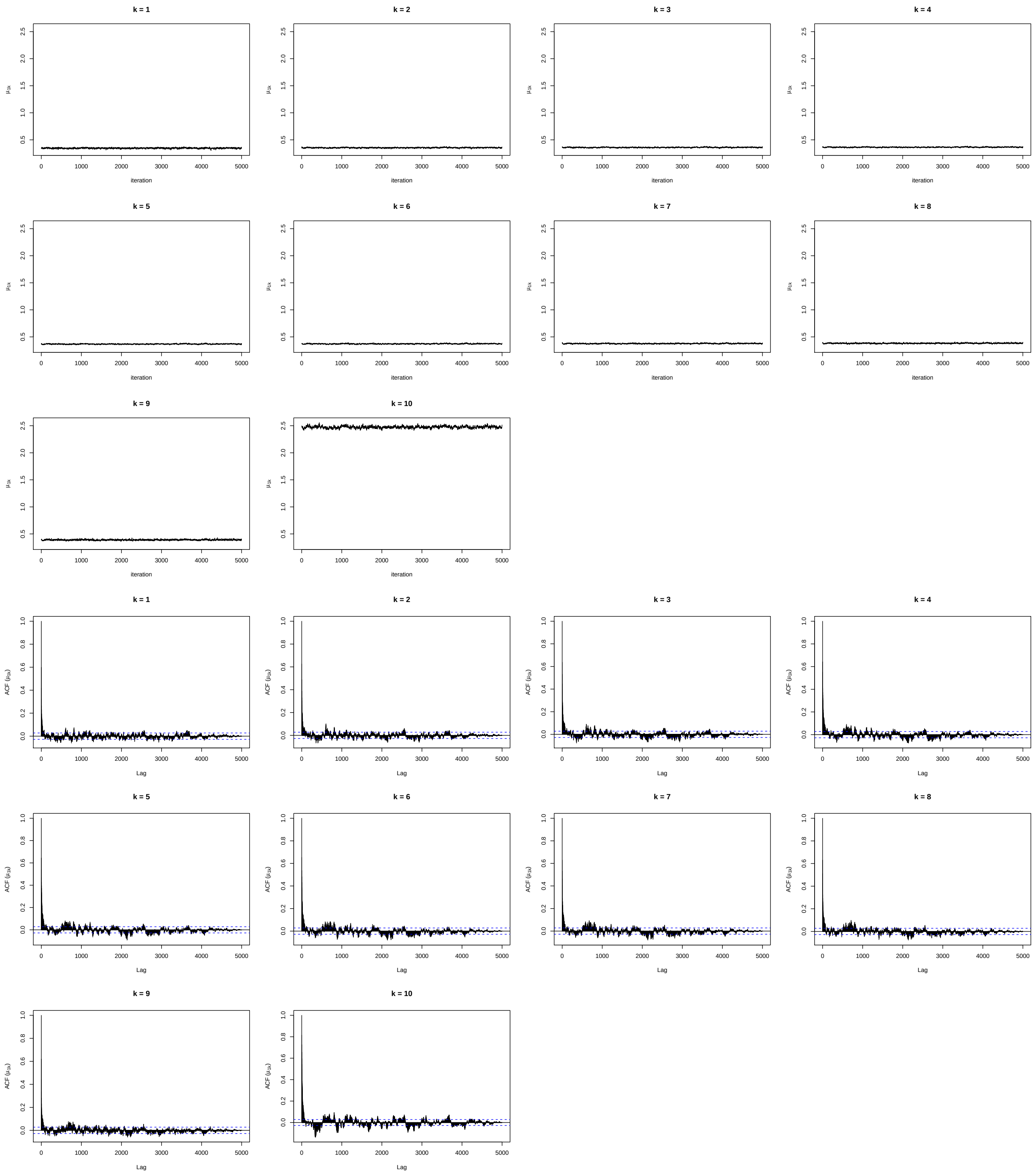

Figure 28: Trace and ACF plots of μ_{1k}. Synthetic Data Containing 5% Active Compounds.

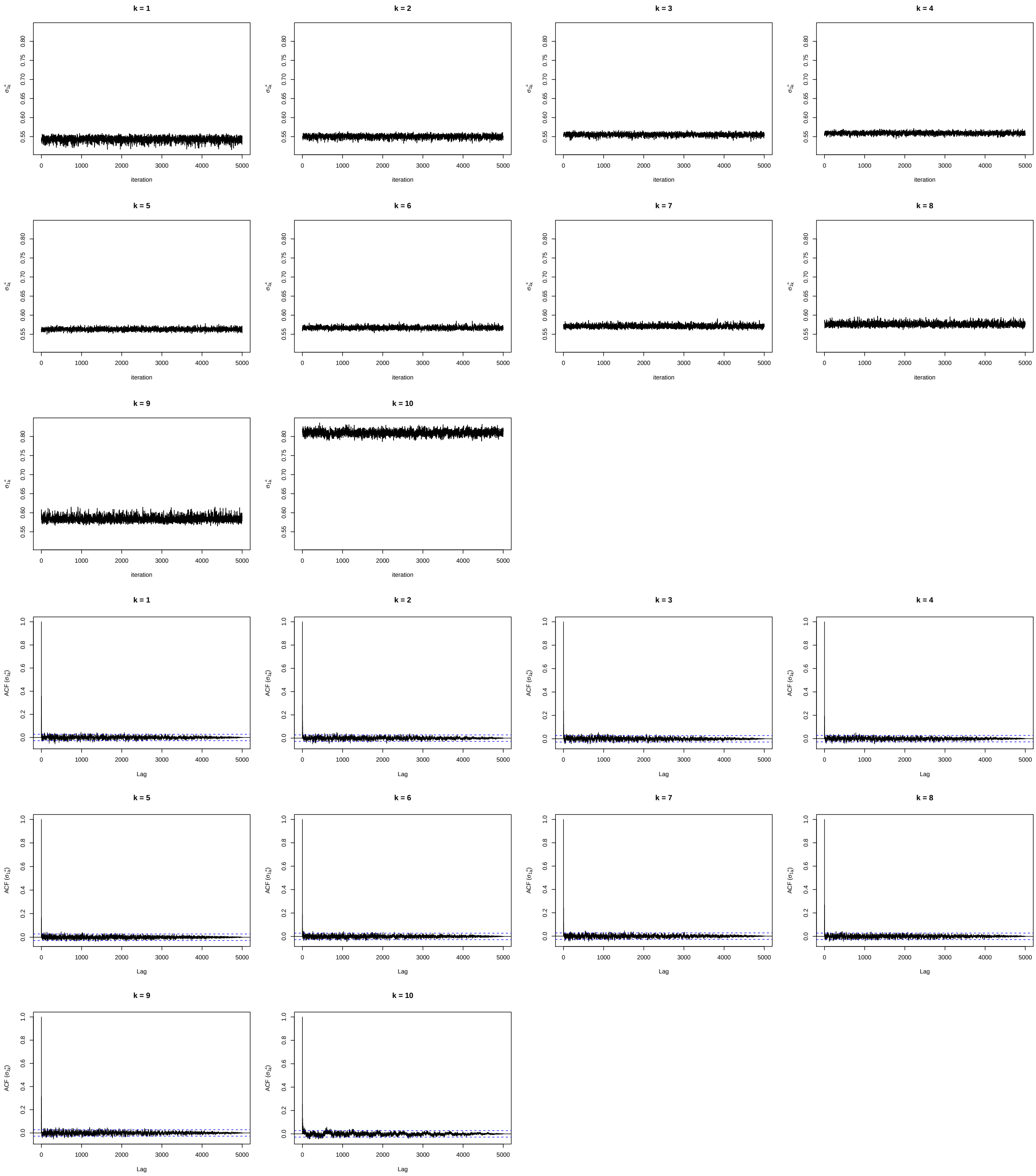

Figure 29: Trace and ACF plots of σ_{1k}^2. Synthetic Data Containing 5% Active Compounds.

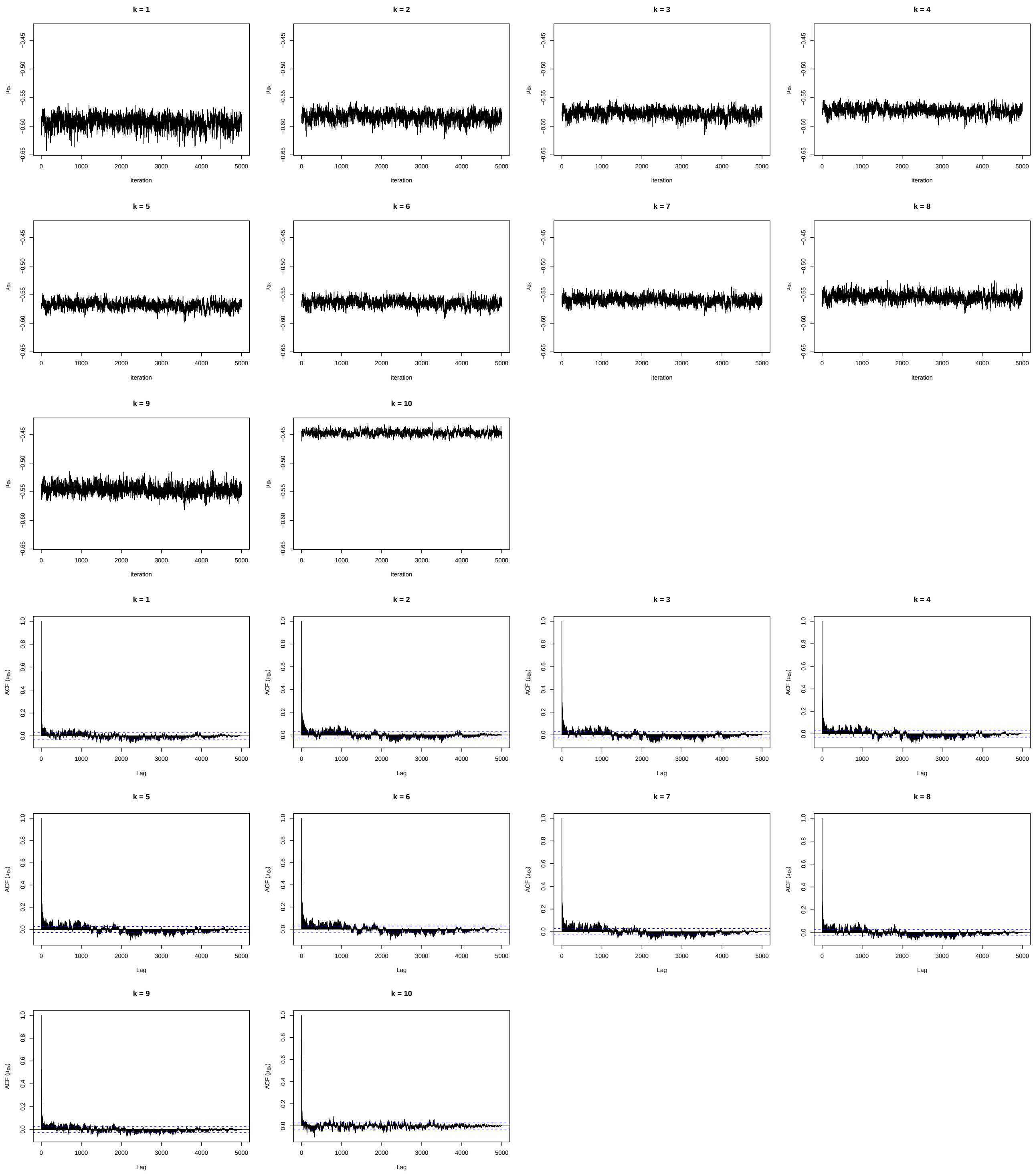

Figure 30: Trace and ACF plots of μ_{0k}. Synthetic Data Containing 5% Active Compounds.

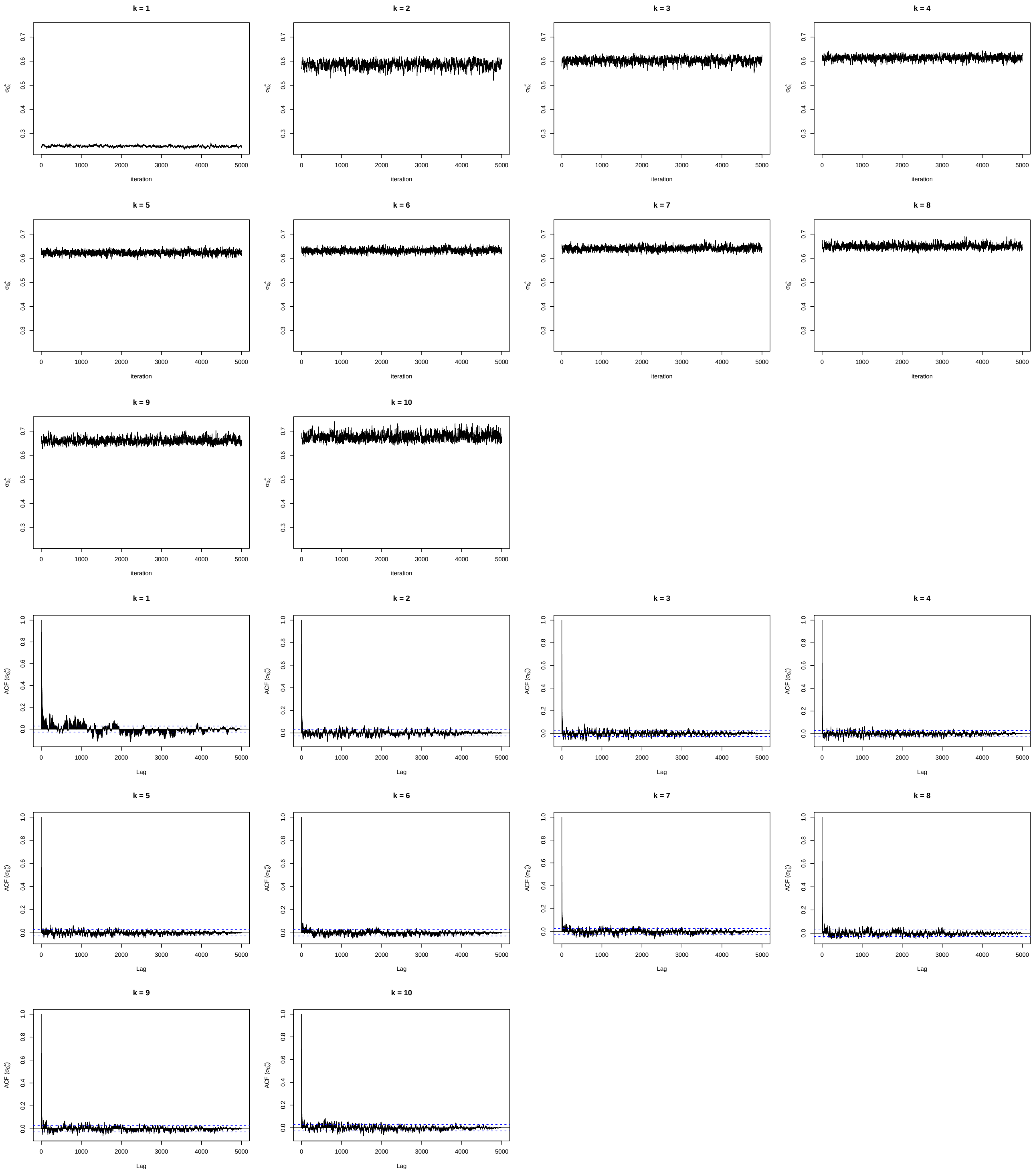

Figure 31: Trace and ACF plots of σ^2_{0k}. Synthetic Data Containing 5% Active Compounds.

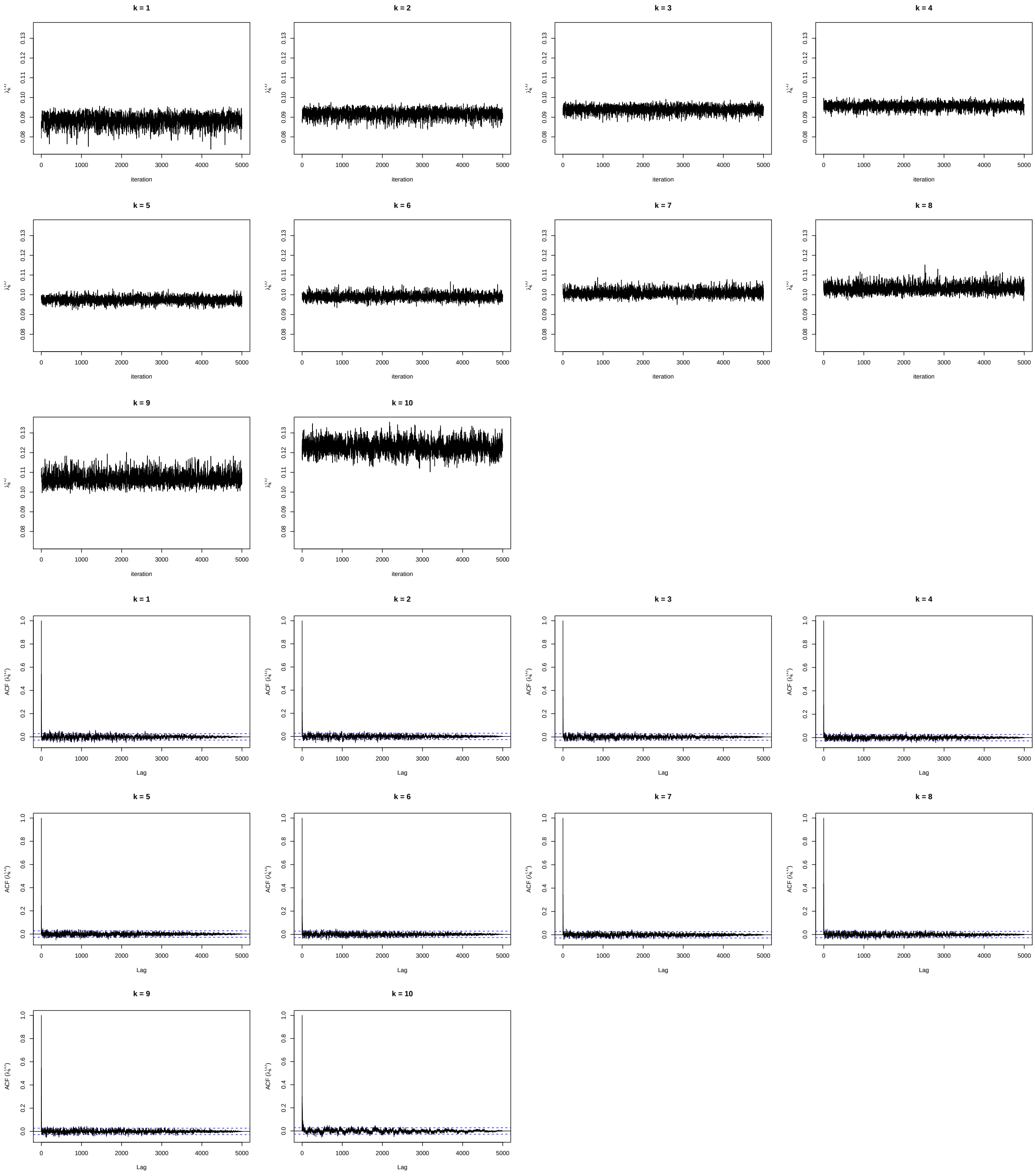

Figure 32: Trace and ACF plots of $\lambda_k^{(1)}$. Synthetic Data Containing 5% Active Compounds.

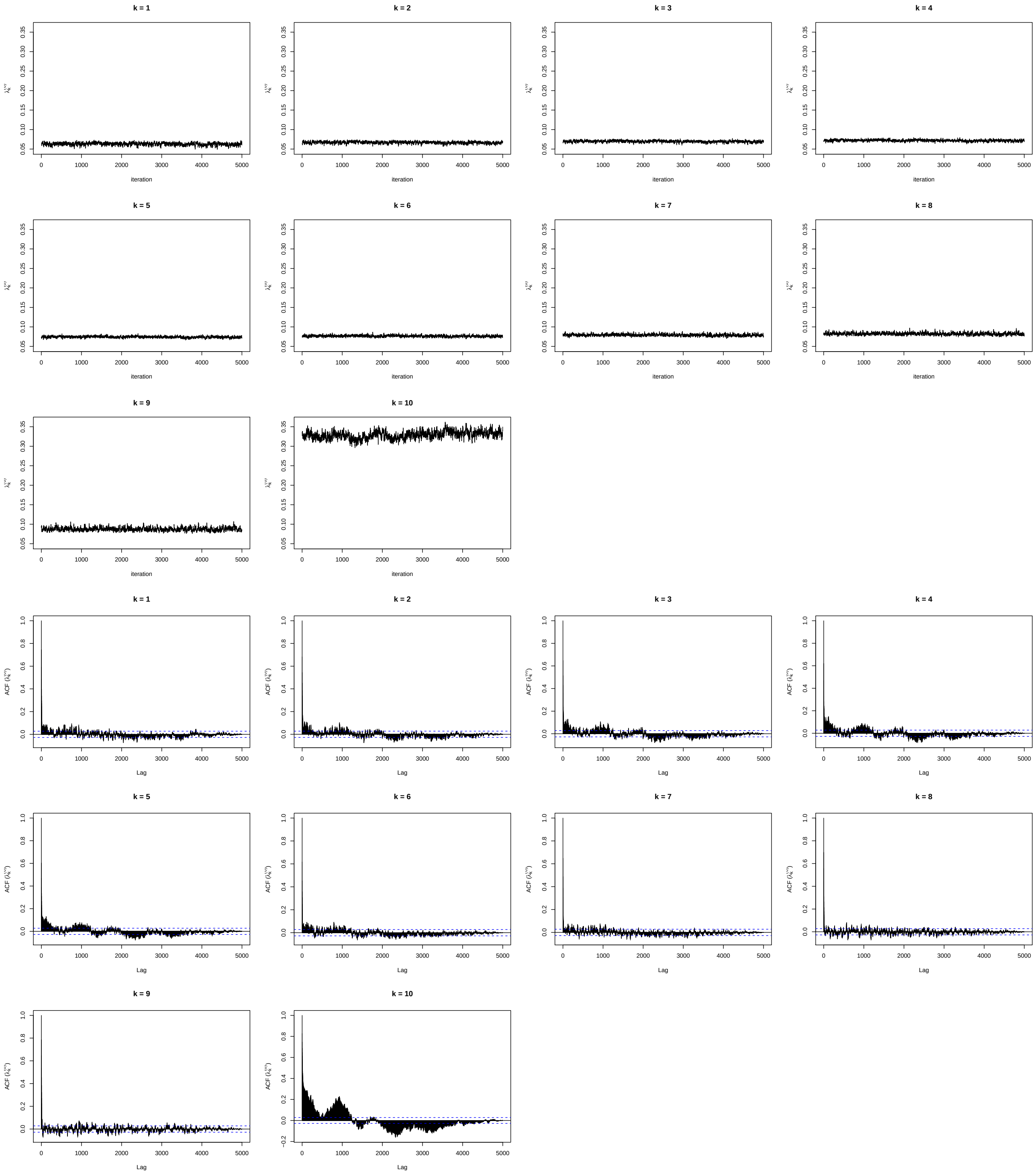

Figure 33: Trace and ACF plots of $\lambda_k^{(0)}$. Synthetic Data Containing 5% Active Compounds.

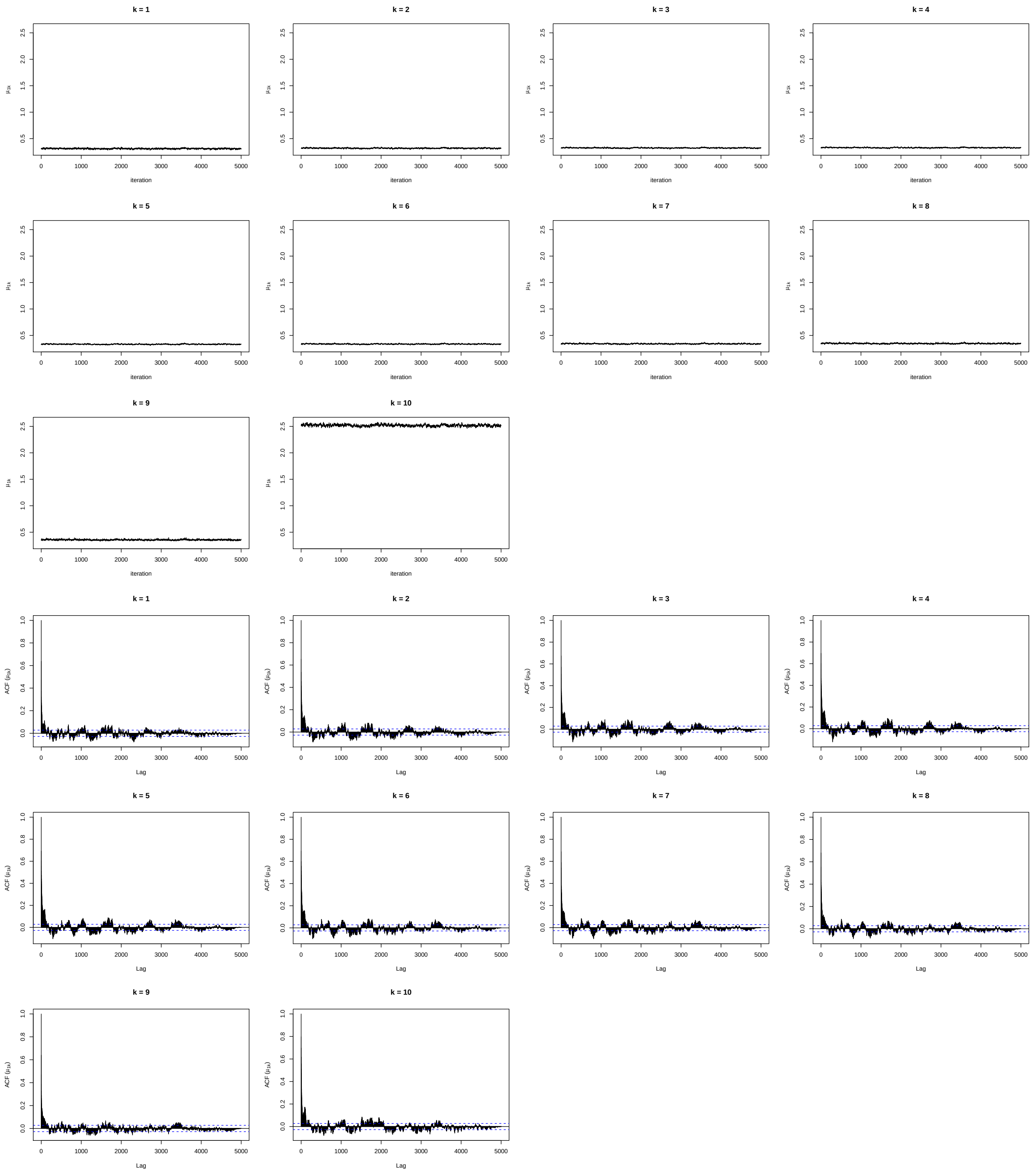

Figure 34: Trace and ACF plots of μ_{1k}. Synthetic Data Containing 1% Active Compounds.

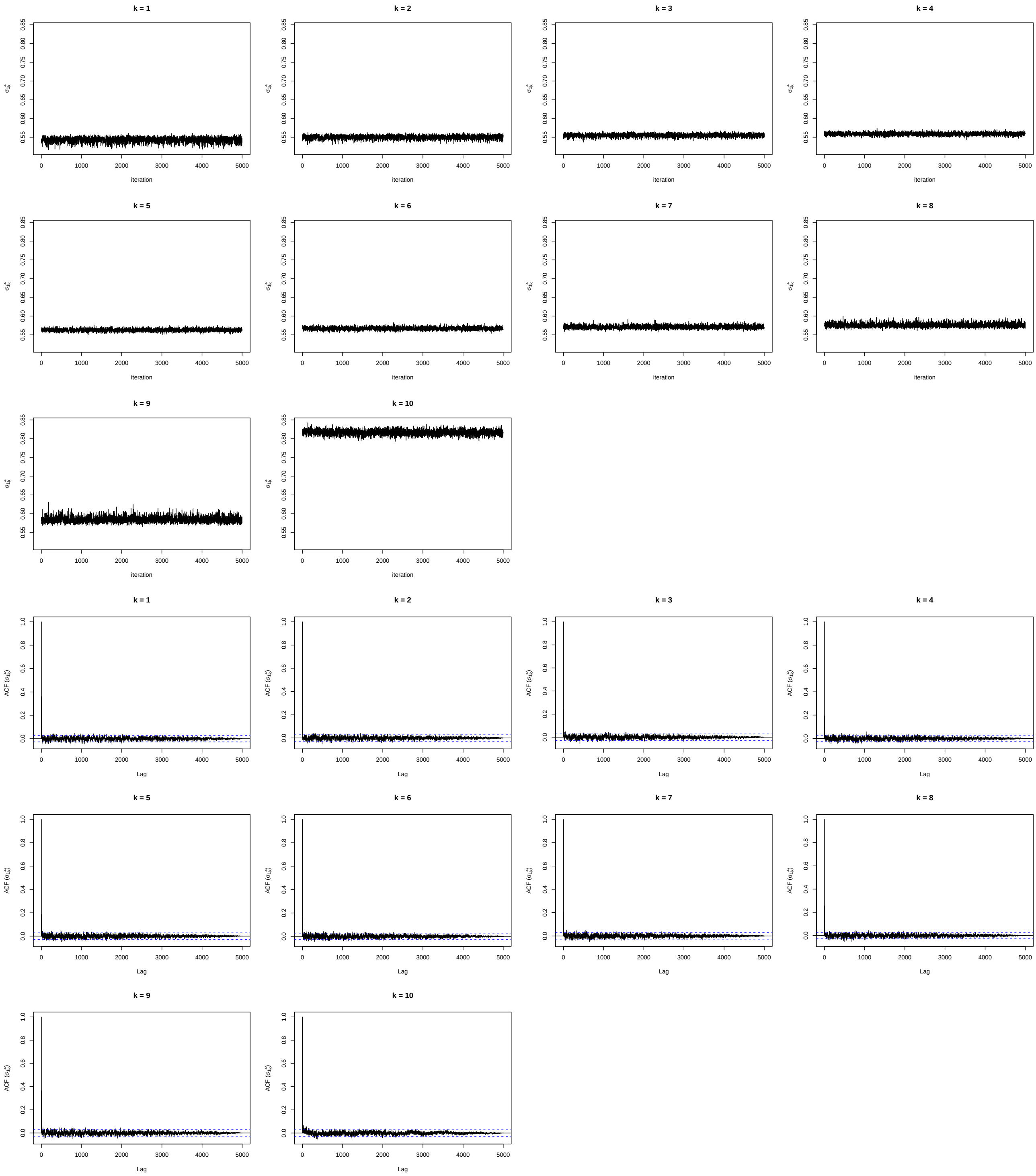

Figure 35: Trace and ACF plots of σ^2_{1k}. Synthetic Data Containing 1% Active Compounds.

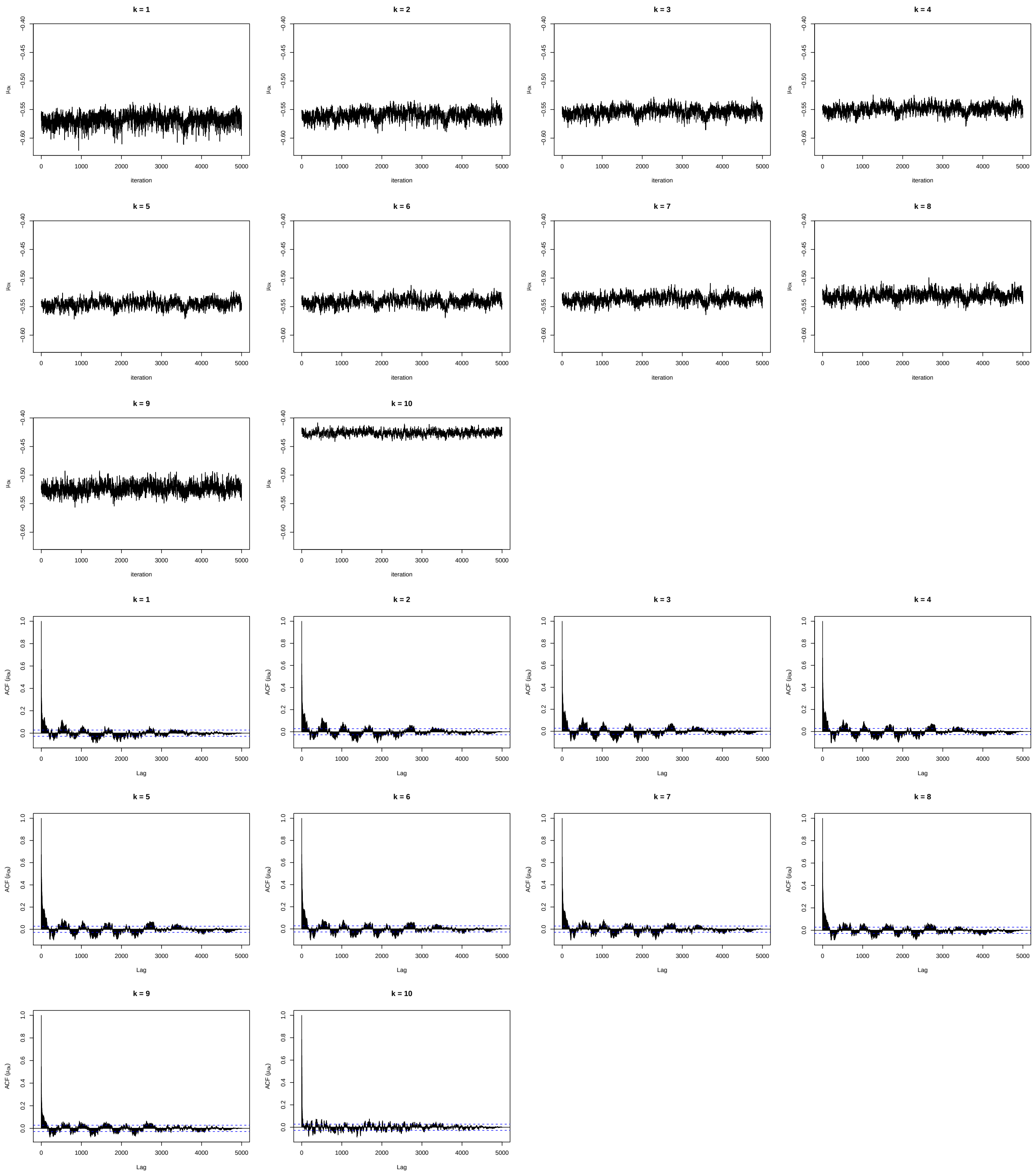

Figure 36: Trace and ACF plots of μ_{0k}. Synthetic Data Containing 1% Active Compounds.

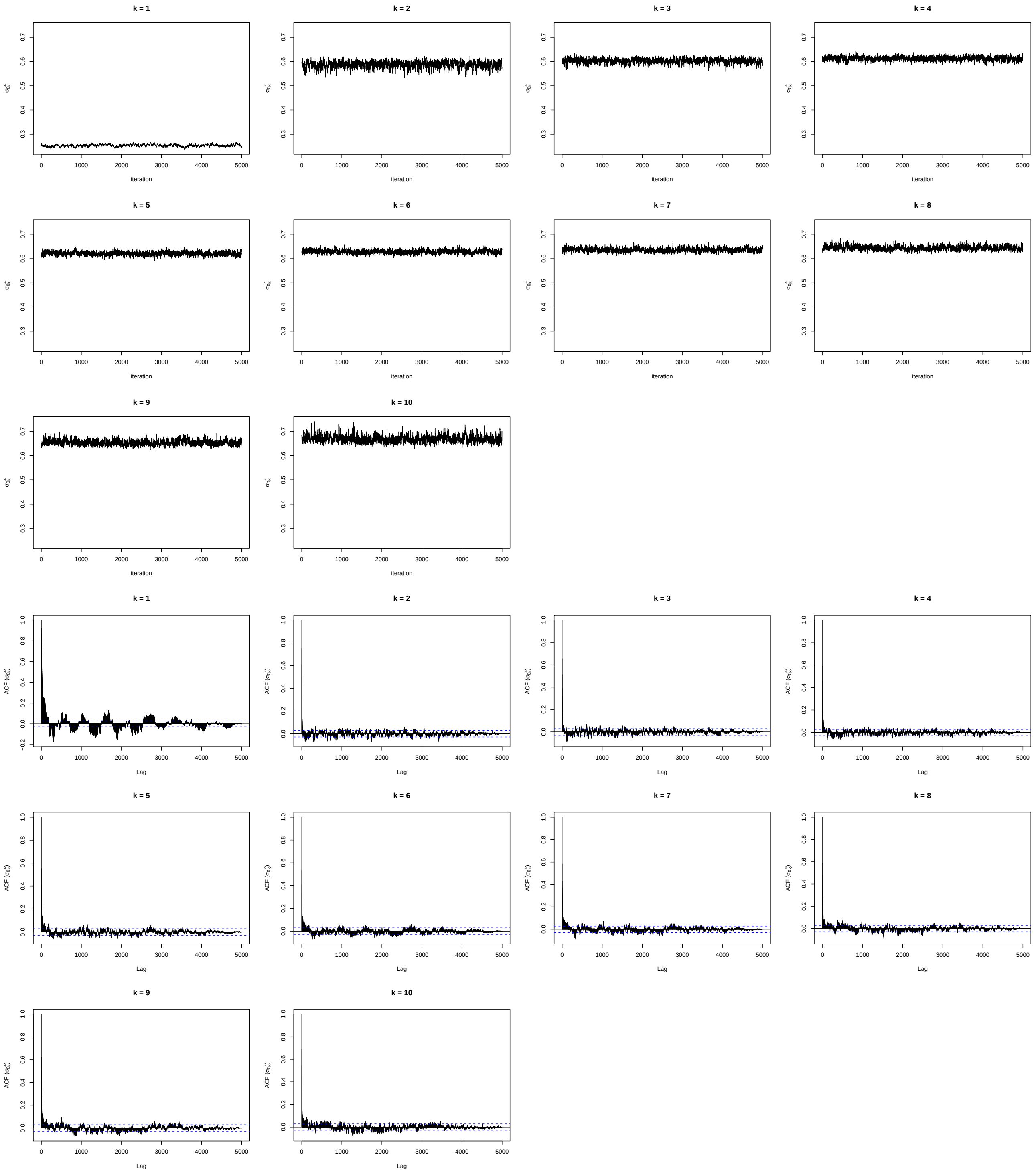

Figure 37: Trace and ACF plots of σ_{0k}^2. Synthetic Data Containing 1% Active Compounds.

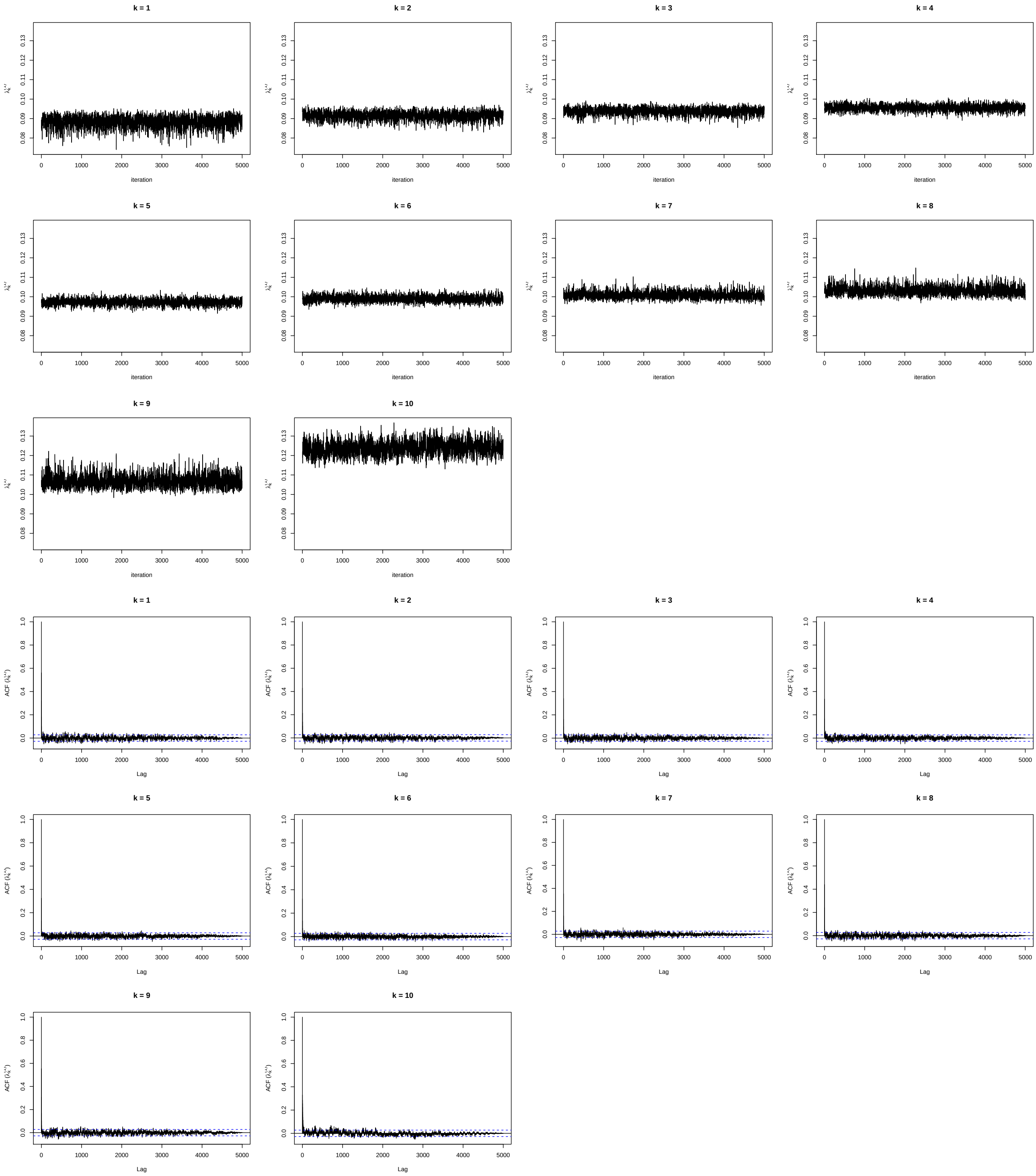

Figure 38: Trace and ACF plots of $\lambda_k^{(1)}$. Synthetic Data Containing 1% Active Compounds.

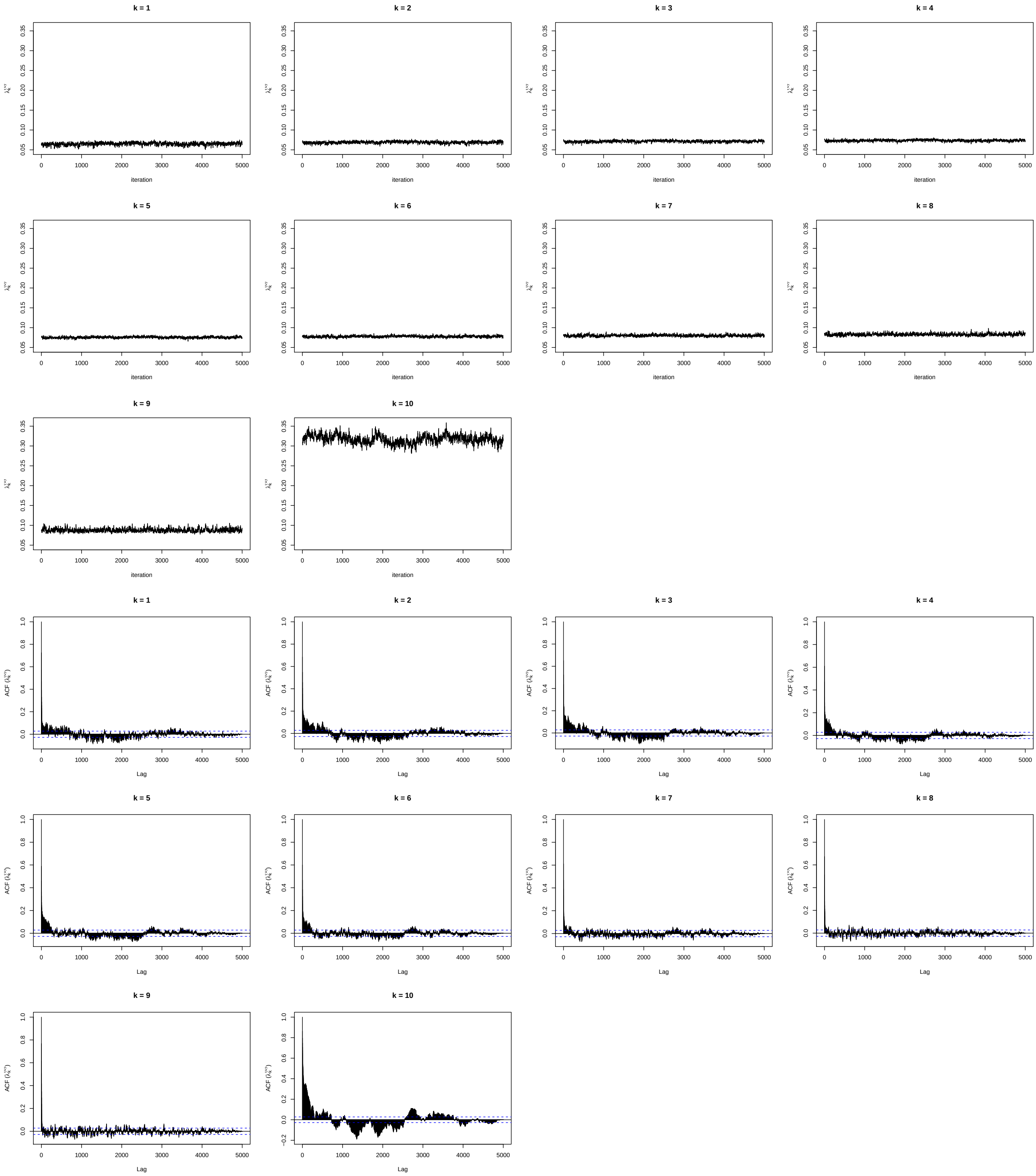

Figure 39: Trace and ACF plots of $\lambda_k^{(0)}$. Synthetic Data Containing 1% Active Compounds.

References

[1] H. Ishwaran and L. F. James. Gibbs Sampling Methods for Stick-Breaking Priors. *Journal of The American Statistical Association*, 96(453):161–173, 2001.

[2] R. E. Kass, B. P. Carlin, A. Gelman, and R. M. Neal. Markov Chain Monte Carlo in Practice: A Roundtable Discussion. *The American Statistician*, 52(2):93–100, 1998.
